# Hydrocephalus in *Nfix^−/−^* Mice Is Underpinned by Changes in Ependymal Cell Physiology

**DOI:** 10.3390/cells11152377

**Published:** 2022-08-02

**Authors:** Danyon Harkins, Tracey J. Harvey, Cooper Atterton, Ingrid Miller, Laura Currey, Sabrina Oishi, Maria Kasherman, Raul Ayala Davila, Lucy Harris, Kathryn Green, Hannah Piper, Robert G. Parton, Stefan Thor, Helen M. Cooper, Michael Piper

**Affiliations:** 1 School of Biomedical Sciences, The University of Queensland, Brisbane 4072, Australia; danyon.harkins@uq.net.au (D.H.); t.harvey1@uq.edu.au (T.J.H.); c.atterton@uq.edu.au (C.A.); i.miller@uq.net.au (I.M.); l.currey@uq.net.au (L.C.); s.oishi@uq.edu.au (S.O.); m.kasherman@unsw.edu.au (M.K.); raul.ayaladavila@uq.net.au (R.A.D.); hpipe5@eq.edu.au (H.P.); s.thor@uq.edu.au (S.T.); 2 Centre for Microscopy and Microanalysis, The University of Queensland, Brisbane 4072, Australia; lucy.harris1@uq.net.au (L.H.); kathryn.green@uq.edu.au (K.G.); r.parton@imb.uq.edu.au (R.G.P.); 3 Institute for Molecular Biosciences, The University of Queensland, Brisbane 4072, Australia; 4 Queensland Brain Institute, The University of Queensland, Brisbane 4072, Australia; h.cooper@uq.edu.au

**Keywords:** ependymal cells, cell morphology, adherens junctions, cilia, hydrocephalus, epithelial adhesion

## Abstract

Nuclear factor one X (NFIX) is a transcription factor required for normal ependymal development. Constitutive loss of *Nfix* in mice (*Nfix^−/−^*) is associated with hydrocephalus and sloughing of the dorsal ependyma within the lateral ventricles. Previous studies have implicated NFIX in the transcriptional regulation of genes encoding for factors essential to ependymal development. However, the cellular and molecular mechanisms underpinning hydrocephalus in *Nfix^−/−^* mice are unknown. To investigate the role of NFIX in hydrocephalus, we examined ependymal cells in brains from postnatal *Nfix^−/−^* and control (*Nfix^+/+^*) mice using a combination of confocal and electron microscopy. This revealed that the ependymal cells in *Nfix^−/−^* mice exhibited abnormal cilia structure and disrupted localisation of adhesion proteins. Furthermore, we modelled ependymal cell adhesion using epithelial cell culture and revealed changes in extracellular matrix and adherens junction gene expression following knockdown of *NFIX*. Finally, the ablation of *Nfix* from ependymal cells in the adult brain using a conditional approach culminated in enlarged ventricles, sloughing of ependymal cells from the lateral ventricles and abnormal localisation of adhesion proteins, which are phenotypes observed during development. Collectively, these data demonstrate a pivotal role for NFIX in the regulation of cell adhesion within ependymal cells of the lateral ventricles.

## 1. Introduction

Within the developing rodent cerebral cortex, radial glia, which are the neural progenitor cells of the brain, produce neurons, glia, and ultimately, ependymal cells. In mice, a subpopulation of radial glia within the lateral ventricles between embryonic days (E) 13.5–15.5 are fated to generate ependymal cells and ventricular-subventricular zone (V-SVZ) adult neural stem cells [1]. Ependymal fated-progenitors proliferate and ultimately differentiate by postnatal day (P) 6 in mice, forming a continuous epithelial sheet of cells that separate the brain parenchyma from the cerebrospinal fluid [1,2,3]. Ependymal cells play numerous key roles in the brain, including mediating signalling to adult neural stem cells through direct cell adhesion or paracrine signalling, contributing to cerebrospinal fluid (CSF) composition and flow, and isolating CSF within the ventricles and spinal cord central column [4,5,6,7,8,9,10,11]. Ependymal development is regulated by networks of transcription factors [6,12,13,14] which facilitate the acquisition of terminal ependymal cell traits [12] and the loss of radial glial traits [15]. One such essential transcription factor is nuclear factor one X (NFIX) [14,16].

*Nfix* knockout (*Nfix^−/−^*) mice exhibit a range of cortical abnormalities, including enlarged ventricles [16,17]. These enlarged ventricles are first apparent at P5 in *Nfix^−/−^* mice and are present in all *Nfix^−/−^* mice by P10 [14,16]. These mice, which die at weaning (~P20), exhibit frank hydrocephalus at this age [16,17]. This phenotype is even more severe in mice lacking both *Nfix* and *Nfib* [16]. Importantly, no evidence of damage or physiology consistent with blockage of the ventricular system or stenosis of the Sylvian aqueduct has been observed in the ventricles of *Nfix^−/−^* mice [14,16]. Moreover, the subcommissural organ of mice lacking *Nfix* and *Nfib* appears morphologically normal and is immunoreactive for Reissner’s fibre [16]. This suggests that the origins of hydrocephalus in *Nfix^−/−^* mice arises from changes in ependymal cells or their precursors such as in other cases of communicating hydrocephalus [5,18,19,20,21,22,23].

NFIX is expressed along the walls of the ventricles at E14 [16]; these are likely neurogenic or gliogenic radial glial populations [1,13]. NFIX expression is maintained along the ventricular walls at E16 and E18, and may include populations of radial glia committed to an ependymal cell fate and immature ependymal cells, respectively [14]. By P0 lateral ventricle cells expressing the ependymal cell marker FOXJ1 express low levels of NFIX. NFIX expression increases over the postnatal period becoming more prominent by P5 and P10, resulting in all FOXJ1^+^ cells strongly expressing NFIX by P15 [14]. Interestingly, *Nfix^−/−^* mice demonstrate reduced levels of FOXJ1 expression at P0 and P5 compared to controls, implicating NFIX in driving *Foxj1* transcription during the early postnatal period [14].

FOXJ1 is a transcription factor required for the acquisition of ependymal cell terminal identity characteristics including cuboidal/columnar cell morphology and cilia formation [12,24]. Loss of FOXJ1 during development is associated with hydrocephalus and results in ependymal cells displaying a reduction of cilia and which lack the expression of terminal ependymal identity markers including S100β [12]. Prior to birth (E18.5) in mice, FOXJ1 is expressed at low levels by ependymal cells [12,25] and increases in expression over the early postnatal period [12]. Although FOXJ1 expression is downregulated in *Nfix^−/−^* mice at P0 and P5, loss of NFIX is not sufficient to prevent FOXJ1 expression in the lateral ventricles. By P15, the expression of FOXJ1 in ependymal cells of *Nfix^−/−^* mice is comparable to controls [14]. Alongside altered FOXJ1 expression, sloughing of the ependyma is observed in the dorsal regions of P10 *Nfix^−/−^* lateral ventricles [14,16]. This suggests that ependymal cell adhesion may be weakened in *Nfix^−/−^* mice. The failure to establish and maintain the ependymal barrier may lead to cells being separated from each other as CSF pressure increases in the lateral ventricles over the early postnatal period [20,26,27]. As such, these phenotypes may be indicative of reduced ependymal adhesion, which may result in ependymal barrier failure similar to the denudation observed in other hydrocephalus models with disrupted NSC and ependymal cell adhesion [6,18,28,29].

To investigate the possibility of an adhesion deficit in *Nfix^−/−^* ependymal cells, we characterised ependymal cell development around the onset of hydrocephalus and examined the adult for changes in the localisation of adhesion proteins at the cell junction. We also used electron microscopy to examine ependymal morphology and cilia structure. Finally, we used epithelial cell culture to model NFIX in ependymal cell adhesion using a NFIX knockdown lentivirus. Through this process we demonstrate a role for NFIX in the modulation of ependymal cell adhesion and posit that NFIX plays a central role in the formation and maintenance of ependymal cell junctions.

## 2. Materials and Methods

### 2.1. Experimental Animals

Two experimental mouse stains were used during this study, namely *Nfix KO* mice and *Nfix^iFOXJ1-GFP^* mice. Both lines were generated on a C57BL/6J background and were housed at the University of Queensland’s Queensland Brain Institute animal facility. Mice were housed and treated in accordance with the Australian Code of Practice for the Care and Use of Scientific Animals. Animals were separated by sex into different boxes prior to sacrifice or breeding. Tamoxifen treated mice were further isolated from corn oil treated siblings to prevent tamoxifen contamination through waste or bedding. Both sexes were used in experiments throughout this study.

### 2.2. Statement of Animal Ethics

All breeding, housing and experiments were conducted according to the Australian Code of Practice for the Care and Use of Scientific Animals and were carried out with approval from the University of Queensland’s Animal Ethics Committee (AEC approval numbers QBI/383/16/NHMRC, 2019/AE000165 and 2020/AE000191).

### 2.3. Nfix KO

Postnatal *Nfix* KO mice were a gift from Professor Richard M. Gronostajski at the University of New York, Buffalo, USA [17]. *Nfix* KO mice have an excision of exon 2 which results in a non-functional *Nfix* allele [17]. For colony maintenance, *Nfix* heterozygous (*Nfix^+/−^*) mice were crossed to *Nfix^+/+^* mice. To generate experimental null animals, male and female heterozygote animals were bred to produce *Nfix^−/−^* mice and littermate controls (*Nfix^+/+^*). *Nfix^−/−^* and *Nfix^+/+^* were collected at P5 and transcardially perfused with either 4% paraformaldehyde (PFA) for immunofluorescence histochemistry (IF-HC) or 2.5% Glutaraldehyde and 2.5% PFA for transmission electron microscopy (TEM). Only *Nfix^+/+^* mice were used as controls for this study as heterozygous *Nfix* mice also exhibit cortical phenotypes [30]. Animals were genotyped using PCR, which produced a 309 base pair (bp) band for the knockout allele and a 213 bp band for the wild-type allele [17]. Primers are included in the supplementary materials.

### 2.4. Nfix^iFOXJ1-GFP^

*Foxj1^CreERt2-GFP^* mice were originally generated by Muthusamy et al. [25]. *Foxj1^CreERt2-GFP^* mice contain a CreERT2 construct tagged with GFP knocked into one allele of the *Foxj1* locus. The CreERT2 knock-in construct prevents transcription of a functional FOXJ1 construct from the knocked in allele. *Foxj1^−/−^* mice are largely postnatal lethal and those that survive exhibit significant hydrocephalus as the result of absence of motile ependymal cilia [12,24]. To avoid undue mortality and hydrocephalus, *Foxj1^CreERt2-GFP^* mice were maintained as heterozygous for the CreERT2-GFP construct. Despite previous publications reporting no overt phenotypes in these heterozygous *Foxj1^CreERt2-GFP/+^* mice [25], we observed an increase in hydrocephalus in this line (data not shown). *Foxj1^CreERt2-GFP/+^* mice were closely monitored for signs of hydrocephalus and culled if mice started to exhibit signs of stress.

*Nfix^iFOXJ1-GFP^* were created specifically for this study by crossing *Foxj1^CreERt2-GFP^* mice with *Nfix^flox/flox^* mice. *Nfix^flox/flox^* mice are phenotypically normal [31,32]. *Nfix^flox/flox^* mice were crossed to *Foxj1^CreERt2-GFP/+^* mice to generate *Nfix^flox/+^;Foxj1^CreERt2-GFP/+^* mice (Figure S1A). To generate experimental animals, *Nfix^flox/+^;Foxj1^CreERt2-GFP/+^* mice were crossed with *Nfix^flox/flox^* mice. A proportion of this cross had the following genotype: *Nfix^flox/flox^;Foxj1^CreERt2-GFP/+^* (Figure S1A; hereafter called *Nfix^iFOXJ1-GFP^*). These mice were used as experimental animals. As with the *Foxj1^CreERt2-GFP/+^* mice, some corn oil and untreated *Nfix^iFOXJ1-GFP^* mice developed signs of hydrocephaly. These mice were immediately culled and were not used for breeding or experiments.

### 2.5. Tamoxifen Treatment

To induce knockout of *Nfix* in *Nfix^iFOXJ1-GFP^* mice, a single dose of tamoxifen dissolved in corn oil was delivered by intraperitoneal injection (IP injection) delivered at 10–12 weeks of age (Figure S1B). *Nfix^iFOXJ1-GFP^* mice were treated with the corn oil vehicle as the control. Experimental mice were isolated by treatment condition (tamoxifen or corn oil) to prevent cross contamination of tamoxifen in bedding from affecting littermates. Doses were given at 130 mg of tamoxifen per kg of body weight or equivalent volume corn oil for controls (5 μL per g of body weight). Mice were closely monitored in the hour following treatment and then were checked twice daily for changes in behaviour or body weight and for signs of stress. Treated mice were sacrificed 7 days post injection (7 dpi) and were transcardially perfused.

### 2.6. Perfusion and Immunofluorescence (IF) Histochemistry

Animals were transcardially perfused with PBS then 25–30 mL of 4% paraformaldehyde (PFA). Once perfused, the skull was partially dissected to gain access to the brain and the head was further post-fixed for 72 h at 4 °C. After post-fixing brains were transferred into PBS + 0.02% sodium azide.

Post-fixed P5 *Nfix KO* and wild-type brains were transferred into histology cassettes and dehydrated via an ethanol series into 70% ethanol before being embedded into paraffin via an ASP3000s Pathcentre (Leica, Wetzlar, Germany). Once processed into paraffin, brains were stored at 4 °C until embedding using a TES Valida embedding station (Medite, Hildesheim, Germany). Once set, processed brains were transferred to a RM2245 semi-automated rotary microtome (Leica, Wetzlar, Germany) and serially sectioned into 6 μm sections. Each brain was serially sectioned caudally until reaching the hippocampus. Once dry, sections were collected in slide boxes and stored in a cool, dry place. For IF, paraffin sections were dewaxed by heating to 65 °C, washing in histolene baths and rehydrated via an ethanol series. Once rehydrated sections were subject to antigen retrieval in pH 6.0 citrate buffer inside an antigen retrieval chamber at 95 °C for 15 min.

Post-fixed corn oil and tamoxifen treated adult *Nfix^iFOXJ1-GFP^* brains were serially sectioned into 50 µm thick sections on a vibratome (Lecia, Wetzlar, Germany) and stored in PBS + 0.02% Sodium azide until use. Sections were mounted in cold PBS to superfrost+ (Thermofisher, Waltham, MA, USA) slides and dried at 37 °C before being subject to antigen retrieval in pH 6.0 citrate buffer inside an antigen retrieval chamber at 95 °C for 15 min.

Post antigen retrieval, both *Nfix KO* and *Nfix^iFOXJ1-GFP^* sections were treated identically. Sections were blocked in 2% Donkey serum, 0.2% Triton X-100 in PBS (blocking buffer) for 2 h before sections were washed with PBS + 0.1% Triton X-100. Sections were incubated overnight at 4 °C with primary antibodies diluted in blocking buffer at concentrations listed in Table 1. The following day, sections were washed with PBS + 0.1% Triton X-100 and incubated for one hour at room temperature with secondary antibodies diluted in blocking buffer at concentrations listed in Table 1. Sections were also counterstained with 4′,6-diamidino-2-phenylindole (DAPI) nuclear marker before being mounted with a coverslip using Dako fluorescence mounting medium (Agilent, Santa Clara, CA, USA).

### 2.7. Cell Culture

MCF7 cells were obtained from Professor Alpha Yap (The University of Queensland’s Institute for Molecular Bioscience, Australia). MCF7 cells are an immortalised epithelial cancer cell line isolated from mammary cancer cells that have previously been used to model epithelial cell adhesion [33]. All experiments were conducted on MCF7 cells between passages 23 and 35. No changes in cell morphology were observed between these passage numbers. For immunostaining, MCF7 cells were grown on 15 mm coverslips placed at the bottom of sterile six-well plates. MCF7 cells were cultured in 2 mL growth media made from 10% Foetal Bovine Serum, 1–2% Penn-Strep, non-essential amino acids + L-glutamate in DPMI media. Wells were seeded at approximately 1 × 10^5^ cells and grown at 37 °C, 5% CO_2_ in a humidified chamber.

### 2.8. Lentiviral Treatment

At approximately 50–60% confluency, MCF7 cells were treated with one of two NFIX shRNA lentiviral constructs or a scrambled RNA lentiviral construct. The scrambled RNA lentivirus construct and the first of two NFIX shRNA lentivirus constructs (NFIX KD1) were gifts from the Francois Guillemot (The Francis Crick Institute, London, UK). The second NFIX shRNA lentivirus construct (NFIX KD2) was a commercially available construct obtained from Mission Sigma (Merck, Kenilworth, NJ, USA). For MCF7 cells grown in six-well plates 1300 µL of growth media was removed from each well before 350 µL of scramble or NFIX knockdown lentivirus was applied depending on the treatment. NFIX KD cells and paired scrambled controls were always grown in the same plates in different wells. All equipment exposed to viral particles was decontaminated with Vircon and bleach. Six-well plates were placed back at 37 °C and allowed to reach 90–100% confluency (approximately 48 h). At 90–100% confluency, coverslips were washed with sterile PBS and fixed with 4% PFA for 20 min. After fixation, coverslips were washed with and stored in sterile PBS at 4 °C until use.

### 2.9. Cell Culture Immunofluorescence (IF)

Coverslips with adhered and 4% PFA fixed MCF7 cells were permeabilised with 0.2% Triton X-100 for 20 min before being washing with PBS. Coverslips were treated with blocking buffer for 1 h at room temperature before being incubated with blocking buffer for 1 h at room temperature. Blocked coverslips were washed with PBS and incubated with primary antibodies diluted in blocking buffer at the concentrations listed in Table 2 for 1 h. Following primary antibodies sections were washed with PBS and further incubated with secondary antibodies diluted in PBS at the concentrations listed in Table 2 for 1 h. Coverslips were counterstained with DAPI and mounted onto slides using Dako fluorescence mounting media (Agilent, Santa Clara, CA, USA).

### 2.10. Immunofluorescence (IF) Imaging

IF conducted on postnatal *Nfix KO* brains, cell culture coverslips and adult *Nfix^iFOXJ1-GFP^* brains were imaged using a spinning diskovery microscope (Nikon, Tokyo, Japan) located at The University of Queensland’s School of Biomedical Sciences imaging facility. Images were taken at 60× using a 60× Apo TIRF oil objective and a Andor Zyla 4.2 Megapixel 10-tap camera (Andor Technology, Belfast, UK). For P5 brains 10 µm thick stacks were taken in 1 µm increments. For cell culture coverslips 14 µm thick stacks were taken in 1 µm increments. For adult brains 15 µm thick stacks were taken in 1 µm increments. Images were taken under identical conditions for each treatment and its paired controls for each antibody, to reduced technical error between replicates. Images were taken as greyscale, pseudocoloured in Fiji (ImageJ, https://imagej.net/software/fiji/downloads, accessed on 13 June 2022) and cropped and resized for figures in Illustrator (Adobe, https://www.adobe.com/au/products/illustrator.html?sdid=B4XQ3QVJ&mv=search&ef_id=EAIaIQobChMIjJnysL-k-QIVd5JmAh0SjgoOEAAYASAAEgLmavD_BwE:G:s&s_kwcid=AL!3085!3!596063181805!e!!g!!adobe%20illustrator!15573721572!137935232544&gclid=EAIaIQobChMIjJnysL-k-QIVd5JmAh0SjgoOEAAYASAAEgLmavD_BwE, accessed on 13 June 2022).

#### 2.10.1. Line Scanning Analysis

Line scanning analysis was performed on samples that had been stained via IF using antibodies against proteins localised to adherens junctions or tight junctions. Images of fluorescence stains were opened in Fiji (ImageJ) and boxes of 150 pixels across by 20 pixels high were drawn perpendicular to cell junctions of adjacent cells with the centre of the box placed directly over the centre of the junction (Figure S2A). Average fluorescence intensity was measured at each pixel using the plot profile function (Figure S2B) and recorded in GraphPad Prism 8 (Graphpad, https://www.graphpad.com/scientific-software/prism/, accessed on 13 June 2022). These measurements were repeated for each sample across >50 different cell junctions per section from 3 replicates for each antibody stain (Figure S2C). The median fluorescence value of the combined measurements from each sample for each antibody stain was then subtracted from each measurement to account for background fluorescence intensity. The mean fluorescence intensity for each point was then plotted minus the background intensity using GraphPad Prism 8 (Figure S2E). The resulting average fluorescence intensity at each point was then plotted in GraphPad Prism 8 (Graphpad). These graphs were used as proxy measures of the distribution of proteins across the cell junction. The highest point of each average fluorescent intensity graph is referred to as peak fluorescence (Figure S2B).

The maximum fluorescence intensity minus the background correction for each measurement was also separately recorded and plotted using GraphPad Prism 8 (Figure S2F) and was used as a proxy measure of total protein present along the cell junction. This measurement was referred to as maximum fluorescence and is not the same measurement as peak fluorescence. Although maximum fluorescence and peak average fluorescence intensity are generated from the same dataset, the brightest point along the cell junction may not necessarily be directly at the centre and may not represent the same value.

#### 2.10.2. Fluorescence Intensity Analysis

Fluorescence intensity analysis was performed against sections of tamoxifen and corn oil treated *Nfix^iFOXJ1-GFP^* lateral ventricles stained with FOXJ1 or NFIX. Images of fluorescence stains were opened in Fiji (ImageJ). The freehand tool was used to draw around FOXJ1^+^ or NFIX^+^ nuclei respectively, and the measure function was used to record the mean grey value. These measurements were repeated 50 times per sample for 3 samples for each treatment condition (tamoxifen and corn oil) and antibody stain. Background fluorescence was additionally measured 5 times per sample in regions of the image where no tissue was present. Average background fluorescence was subtracted from each fluorescence intensity measurement to equalise background between samples.

### 2.11. Transmission Electron Microscopy (TEM)

TEM was performed on P5 *Nfix KO* brains that were transcardially perfused with PBS and then 15–20 mL 2.5% PFA, 2.5% glutaraldehyde in PBS. Once perfused the skulls of *Nfix* KO mice were partially dissected to expose brain tissue before the head was further post-fixed in 2.5% PFA, 2.5% glutaraldehyde in PBS for another 72 h. Brains were then extracted and stored in PBS + 0.02% sodium azide. Brains were sectioned into 200 µm thick sections using a vibratome (Leica Wetzlar, Germany), and 2 mm by 2 mm regions of interest were cut around the lateral ventricles of depth matched sections of *Nfix^+/+^* and *Nfix^−/−^* sections. Sections were dehydrated and embedded in Epon resin using a Pelco Biowave (Ted Pella, Redding, CA, USA) according to the manufacturer’s instructions. Samples were washed in sodium cacodylate buffer and then placed in 1% osmium tetroxide in 0.1 M sodium cacodylate buffer before being dehydrated in a graded series of ethanol. Samples were then gradually infiltrated with increasing concentrations of Epon, then placed in fresh 100% Epon in the lid of an inverted Eppendorf and polymerised in an oven for 48 h at 60 °C. A diamond knife was used to cut sections at 70 nm. Sections were mounted on a copper grid, contrasted with uranyl acetate and lead and imaged on a JSM1011 microscope (JEOL, Tokyo, Japan) housed at The University of Queensland’s Centre for Microscopy and Microanalysis.

### 2.12. RT^2^ qPCR Arrays

MCF7 cells for qPCR analysis were grown in six-well plates as for IF but without coverslips. At approximately 90–100% confluency, growth media was removed and wells were washed with sterile PBS. Then, 0.5 mL of 0.05% trypsin was applied to each well at 37 °C for 3 min in a humidified chamber to lift cells from the plate. Next, 1.5 mL growth media was used to inactivate trypsin before the contents of each well were transferred to 15 mL falcon and spun down at 200 g for 5 min. The supernatant was carefully removed from each sample and the cell pellets were stored in the falcon tubes at −80 °C overnight. The following day the samples were removed from the freezer and placed on ice before mRNA was extracted from cells using a miRNeasy Mini kit (QIAGEN, Hilden, Germany). After mRNA was extracted, it was converted into cDNA using the “RT^2^ First Stand” kit (QIAGEN, Hilden, Germany) before being stored at −20 °C until use. Samples were removed from −20 °C, thawed and placed on ice. Master mix was prepared as described in the RT^2^ qPCR profiler array manual for format E. Samples were loaded into the provided 384 well plate by pipette using the loading guides provided. A seal was used to cover the plate and was pressed hard to ensure no air was trapped under the seal. Next, 384 well plates were spun at 15,000 RPM for 2 min before loading into a QuantStudio 7 real-time PCR cycler (Thermofisher, Waltham, MA, USA). Samples were run using the preloaded program for fast-SYBR green and the output exported to excel. Relative fold change in gene expression was calculated using the provided excel template and plotted in excel. RT^2^ qPCR fold change was compared by qPCR using the excel spreadsheet provided with the RT^2^ qPCR array (QIAGEN, Hilden, Germany).

### 2.13. Hematoxylin

50 µm sections of post-fixed tamoxifen and corn oil treated *Nfix^iFOXJ1-GFP^* brains were prepared using a vibratome as described above (Section 2.6). Sections were mounted to superfrost+ (ThermoFisher, Waltham, MA, USA) slides in cold PBS and dried for 10–15 min at 37 °C. Sections were rehydrated in tap water for 1 min before immersion in hematoxylin solution for 2 min. Slides were gently rinsed under running tap water for 1 min to remove excess hematoxylin before transferring to an ethanol dehydration series into 100% ethanol. Slides were washed twice in xylene for 5 min each and mounted in DPX mounting media using a coverslip.

### 2.14. Light Microscopy

Images of hematoxylin treated brain sections were taken on an Aperio XT Brightfield Automated Slide Scanner (Leica, Wetzlar, Germany). Images were taken at 20× magnification. Images were opened in Imagescope (Aperio, https://www.leicabiosystems.com/en-au/digital-pathology/manage/aperio-imagescope/, accessed on 13 June 2022) and converted into the tif file format for analysis in Fiji (ImageJ). Hematoxylin images of tamoxifen and corn oil treated *Nfix^iFOXJ1-GFP^* were analysed in Fiji (ImageJ).

### 2.15. Statistical Analysis

Cell counts, maximum fluorescence intensity, fluorescence intensity, and lateral ventricle area measurements were compared between experimental and control groups using a two-tailed students *t*-test assuming equal variance with a threshold of significance of 0.05. All calculations were performed in Graphpad Prism 8 (Graphpad). Cell count comparisons were performed between experimental and control groups with an n of 3. Maximum fluorescence intensity was compared between experimental groups and control groups using a two-tailed student *t*-test with an n of 150, taken from 3 groups of 50 measurements from 3 experimental and control samples respectively. Fluorescence intensity was compared between experimental groups and control groups using a two-tailed student *t*-test with an n of 150, taken from 3 groups of 50 measurements from 3 experimental and control samples respectively. RT^2^ qPCR fold change was compared by qPCR using the excel spreadsheet provided with the RT^2^ qPCR array (QIAGEN, Hilden, Germany). Comparisons of ventricular area obtained from hematoxylin measurements were conducted in GraphPad Prism 8 using an n of 7 tamoxifen and corn oil treated *Nfix^iFOXJ1-GFP^* mice.

## 3. Results

### 3.1. Nfix^−/−^ Mice Demonstrate Disrupted Junctional Localisation of Ependymal Adhesion Proteins and Abnormal Ependymal Cell Morphology

#### 3.1.1. *Nfix^−/−^* Mice Have Reduced Junctional Localisation of Key Adhesion Proteins

Previous studies on *Nfix^−/−^* ependymal cells have reported sloughing of the dorsal ependyma from the walls of the lateral ventricles [14]. As ependymal sloughing is a phenotype commonly associated with impaired ependymal cell adhesion [6,18,28,29,34], we sought to investigate the distribution of key adherens and tight junction proteins across the cell junctions of *Nfix^−/−^* ependymal cells Because hydrocephaly first becomes evident in *Nfix^−/−^* mice at P5, this timepoint was chosen for analysis [14]. To examine the junctional distribution of key tight junction (aPKCζ and ZO-1) and adherens junction (β-Catenin and P120-Catenin) proteins between adjacent ependymal cells we performed IF analysis, imaging and subsequently conducted line scanning analysis (see Materials and Methods). Ependymal cells were identified by co-labelling with either FOXJ1 or vimentin and analysis was performed on ependymal cells from the central regions of depth matched P5 *Nfix^+/+^* and *Nfix^−/−^* mouse lateral ventricles. This analysis yielded both average and maximum fluorescence intensity plots.

**Figure 1:**
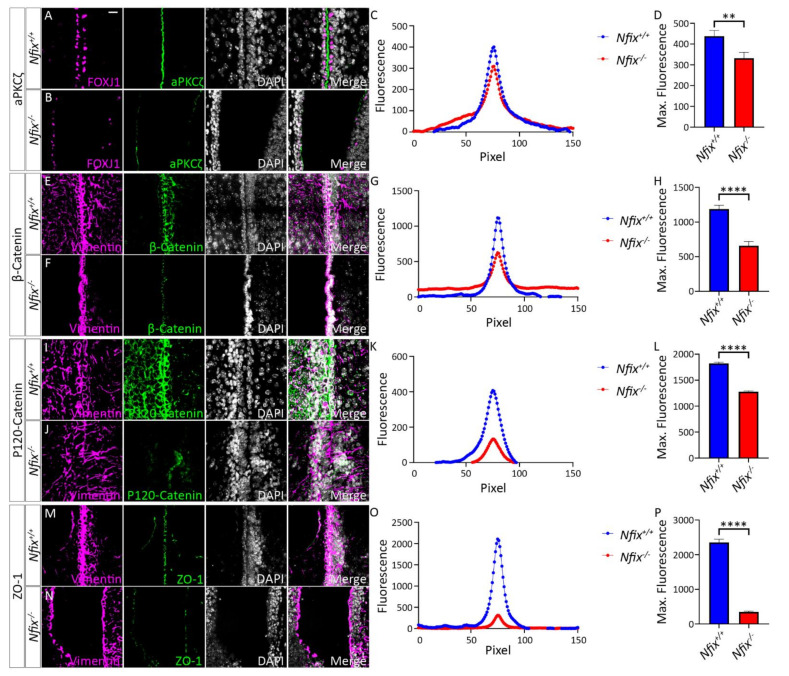
P5 *Nfix^−/−^* mice demonstrate abnormal localisation of key adhesion proteins to the cell junction. (**A**,**B**,**M**,**N**) Images of brain lateral ventricles from P5 *Nfix^+/+^* (**A**) and *Nfix^−/−^* (**B**) mice marked with antibodies against ependymal marker FOXJ1 (**A**,**B**) or Vimentin (**M**,**N**) (purple), tight junction protein aPKCζ (**A**,**B**) or ZO-1 (**M**,**N**) (green) and nuclear marker DAPI (grey) displayed separately and merged for each genotype. (**C**,**O**) Average fluorescence intensity of aPKCζ (**C**) and ZO-1 (**O**) across the cell junctions of *Nfix^−/−^* (red) and *Nfix^+/+^* (blue) ependymal cells. The peak fluorescence intensities of aPKCζ and ZO-1 are lowered in *Nfix^−/−^* mice compared to controls. (**D**,**P**) Maximum fluorescent intensities of aPKCζ (**D**) and ZO-1 (**P**) across the cell junctions of *Nfix^−/−^* (red) and *Nfix^+/+^* (blue) ependymal cells. Maximum fluorescent intensity of aPKCζ is significantly reduced in *Nfix^−/−^* mice compared to controls. (**E**,**F**,**I**,**J**) Images of brain lateral ventricles from P5 *Nfix^+/+^* (**E**,**I**) and *Nfix^−/−^* (**F**,**J**) mice marked with antibodies against ependymal marker vimentin (purple), adherens junction protein β-Catenin (**E**,**F**) or P120-Catenin (**I**,**J**) (green) and nuclear marker DAPI (grey) displayed separately and merged for each genotype. (**G**,**K**) Average fluorescent intensities of β-Catenin (**G**) and P120-Catenin (**K**) across the cell junctions of *Nfix^−/−^* (red) and *Nfix^+/+^* (blue) ependymal cells. The peak fluorescence of β-Catenin and P120-Catenin are lowered in *Nfix^−/−^* mice compared to controls. (**H**,**L**) Maximum fluorescent intensities of β-Catenin (**F**) and P120-Catenin (**L**) across the cell junctions of *Nfix^−/−^* (red) and *Nfix^+/+^* (blue) ependymal cells. Maximum fluorescent intensity of β-Catenin is significantly reduced in *Nfix^−/−^* mice compared to controls. ** *p* < 0.01, **** *p* < 0.0001 two-tailed *t*-test. Graphs depict mean ± s.e.m. from three samples per genotype with an n of 150 junctions. Scale bar (**A**) represents 20 µm.

Previous studies revealed sloughing of the ependymal layer of the lateral ventricles in P10-P20 *Nfix^−/−^* mice [14]. Based upon these findings, we hypothesised that junctional localisation of aPKCζ, β-Catenin, P120-Catenin and ZO-1 would be significantly reduced in P5 *Nfix^−/−^* mice. Consistent with this, IF analysis via line scanning across the cell junction revealed reduced peak fluorescence in average fluorescence intensity plots as well as significantly reduced maximum fluorescence in maximum fluorescence intensity plots in KO mice in comparison to controls for aPKC (Figure 1A–D), β-Catenin (Figure 1E–H), P120-Catenin (Figure 1I–L) and ZO-1 (Figure 1M–P).Together these point to an adhesion deficit in *Nfix^−/−^* ependymal cells.

#### 3.1.2. *Nfix^−/−^* Ependymal Cells Demonstrate Abnormal Morphology

**Figure 2:**
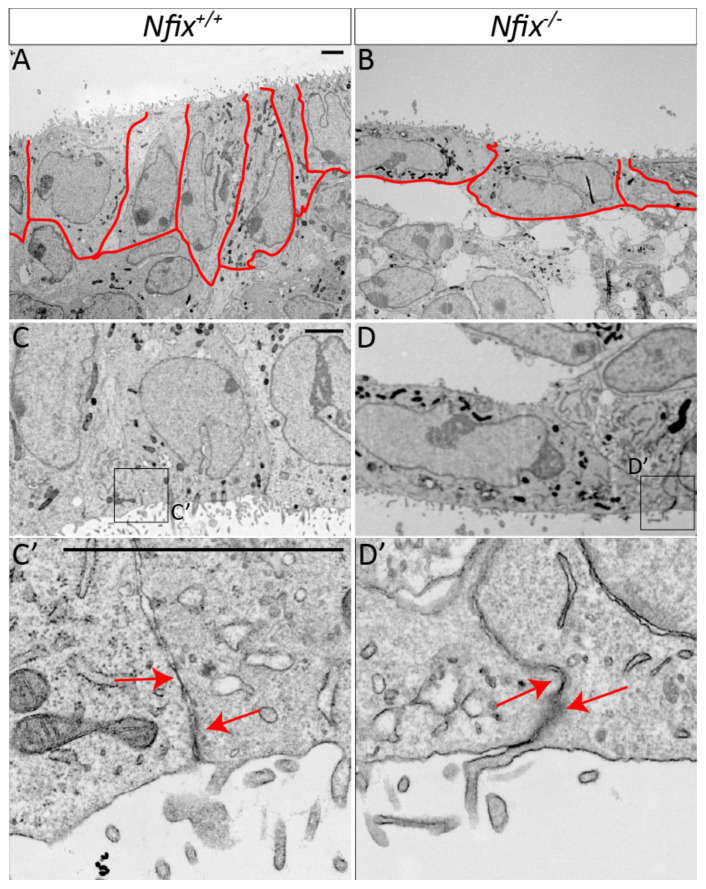
TEM of *Nfix^−/−^* ependymal cells reveal abnormal ependymal cell morphology. (**A**,**B**) Overviews of P5 *Nfix^+/+^* (**A**) and *Nfix^−/−^* (**B**) lateral ventricles depict ependymal cells lining the surface of the ventricles. (**A**) P5 *Nfix^+/+^* ependymal cells are shown to have a columnar appearance outlined in red. (**B**) In contrast to the columnar appearance of controls, *Nfix^−/−^* ependymal cells have a rounded appearance as outlined in red. (**C**,**D**) Images depicting a low magnification view of P5 *Nfix^+/+^* (**C**) or *Nfix^−/−^* (**D**) ependymal cells including one or more ependymal cell-to-cell junction. (**C’**,**D’**) Images contained in boxes (**C’**,**D’**) are enlarged and displayed at higher magnification in panels (**C’**,**D’**). Red arrows highlight the cell junction. P5 *Nfix^−/−^* ependymal cells demonstrated reduced protein density along the cell junctions of adjacent ependymal cells compared to controls. Scale bars (**A**,**C**,**C’**) represents 2 µm.

Adherens and tight junctional proteins play a central role in maintaining epithelial cell shape and function. Given the abnormal localisation of adherens junction proteins in *Nfix^−/−^* ependymal cells we sought to determine if there were morphological changes in these cells using transmission electron microscopy (TEM). We posited that ependymal cell shape and protein density at cell junctions would be different in *Nfix^−/−^* mice. In line with our hypothesis, the morphology of *Nfix^−/−^* ependymal cells at P5 was altered in comparison to controls. Whereas wild-type ependymal cells were columnar, ependymal cells in the mutant lacked this columnar morphology, and instead were more rounded (Figure 2A,B), similar to the cell shape associated with these cells at earlier time-points of development [2]. The lack of columnar characteristics, allied with the aberrant characterise of adherens/tight junction proteins to cell junctions, hints at abnormal cell-cell interactions between ependymal cells in *Nfix^−/−^* mice. Consistent with this, we also observed a reduction in protein density along the cell junctions of *Nfix^−/−^* ependymal cells when compared to controls (Figure 2C’,D’).

**Figure 3:**
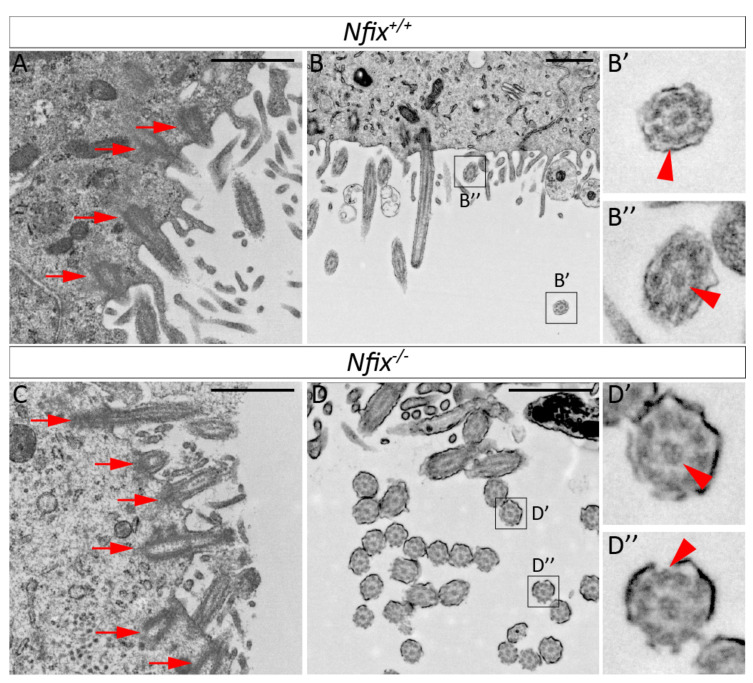
TEM of *Nfix^−/−^* ependymal cells reveal abnormal cilia structure. (**A**,**C**) High magnification images of *Nfix^+/+^* (**A**) and *Nfix^−/−^* (**C**) ependymal cilia anchors depicted by red arrows. No visual differences in cilia anchor structure or protein density could be identified. (**B**,**D**) Images containing ependymal cilia cross sections from P5 *Nfix^+/+^* (**B**) and *Nfix^−/−^* (**D**) mouse lateral ventricles. (**B’**,**B”**) *Nfix^+/+^* ependymal cilia cross sections show the 9 + 2 motile cilia structure with 9 pairs (**B’**, red arrowhead) of microtubules around the outside and 2 (**B”**, red arrowhead) in the centre. (**D’**,**D”**) P5 *Nfix^−/−^* ependymal cells demonstrated structural differences which deviated from the traditional 9 +2 structure including the presence of additional microtubules in the centre (**D’**, red arrowheads) and/or the presence of microtubule triplets around the outer edge (**D”** red arrowhead). Every cilia cross section examined in P5 *Nfix^−/−^* ependymal cells had one or more additional microtubules present along the outer edge or the centre. Scale bars (**A**–**D**) represents 1 µm.

TEM also offers the opportunity to assess cilia structure. Unlike cell-cell junctions, we did not observe significant differences in the protein density or structure at the base of ependymal cilia between controls and mutant, suggestive of normal localisation of actin and intermediate filament proteins to the cilia of *Nfix^−/−^* ependymal cells (Figure 3A,C). We did, however, observe subtle structural alterations in the cilia of *Nfix*-deficient ependymal cells. Cilia from wild-type controls exhibited the classic 9 + 2 structure of microtubules within the ciliary shaft. In contrast, *Nfix^−/−^* ependymal cells regularly exhibited additional microtubules, either 10 + 2, or 9 + 3 (Figure 3D,D’,D”). This suggests that ciliary function may be impaired in *Nfix^−/−^* ependymal cells. Given the essential nature of cilia to ependymal cell function [3,5,6,35,36,37,38], it is possible that this phenotype also contributes to the hydrocephalus seen in *Nfix*-deficient mice.

#### 3.1.3. *Nfix^−/−^* Ependymal Cells Retain Expression of PAX6

**Figure 4:**
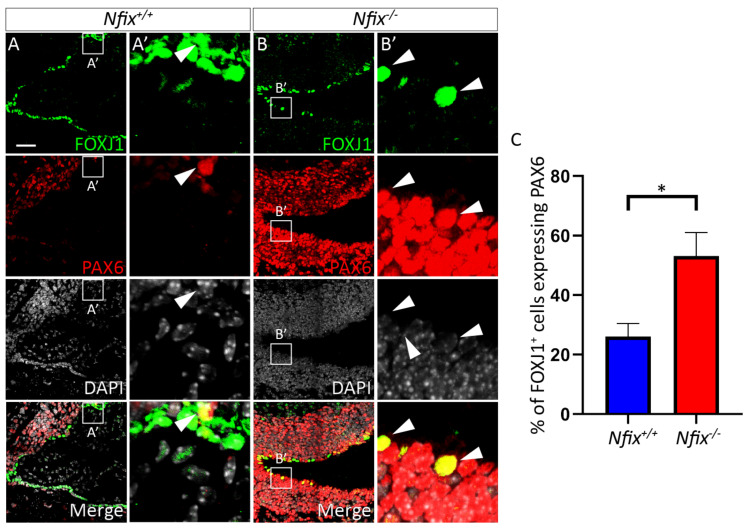
P5 *Nfix^−/−^* ependymal cells have increased expression of PAX6. (**A**,**B**) Images of lateral ventricles from P5 *Nfix^+/+^* (**A**) and *Nfix^−/−^* (**B**) mice stained with antibodies against ependymal cell marker FOXJ1 (green), radial glial cell marker PAX6 (red) and nuclear marker DAPI (grey), displayed separately and merged. (**A’**,**B’**) Enlarged images of regions depicted in white boxes **A’** and **B’** are shown in panels (**A’**,**B’**). White arrowheads depict FOXJ1^+^/PAX6^+^ cells. (**C**) Panel displays the proportion of FOXJ1^+^ ependymal cells also expressing PAX6 in *Nfix^−/−^* mice and controls. A significantly higher proportion of P5 FOXJ1^+^ *Nfix^−/−^* ependymal cells displayed PAX6 expression compared to controls. * *p* < 0.05, two-tailed *t*-test. Graphs depict mean ± s.e.m. from three samples per genotype, 50 nuclei per sample. Scale bar (**A**) represents 50 µm.

The lack of columnar ependymal shape postnatally suggests a delay in the maturation of these cells in *Nfix^−/−^* mice, as ependymal committed radial glia have a rounded appearance and ependymal cells progressively acquire a columnar cell shape during the postnatal period [2]. Indeed, a delay in differentiation of neural progenitor cells has been observed in the neocortex, hippocampus and cerebellum of *Nfix^−/−^* mice [39,40,41]. To determine if ependymal cells of the lateral ventricles were also delayed in their differentiation/maturation, we performed co-IF analysis on P5 brain tissue using the ependymal cell marker FOXJ1, and the radial glial marker PAX6. In the control, FOXJ1 expression was evident in ependymal cells lining the lateral ventricles, and the expression of PAX6 was predominantly limited to cells within the parenchyma. Indeed, only a small percentage of ependymal cells retained the expression of PAX6 at this age in the wild-type (Figure 4A,C). In the mutant, however, the expression of PAX6 was more widespread around the ventricular/subventricular zone (Figure 4A,B), in line with previous reports [42]. Critically, significantly more FOXJ1-expressing cells were also positive for PAX6 (Figure 4B,C), indicative of delayed development of these cells.

### 3.2. Reduced Expression of NFIX in Epithelial Cells Is Associated with Abnormal Localisation of Key Adhesion Proteins

To further probe the molecular and transcriptomic means by which NFIX may regulate cellular adhesion of ependymal cells, we utilised an in vitro epithelial cell model (MCF7 cells). These cells maintain adhesion and tight junctions akin to that seen in ependymal cells [3,43]. To alter NFIX expression in these cells we used two different lentiviral vectors containing shRNA specific for *NFIX* (NFIX KD1 or NFIX KD2; see Materials and Methods). A scrambled vector was used as the control. Both knockdown constructs reduced *NFIX* mRNA expression by approximately 70% (Figure S3). Furthermore, both vectors produced identical phenotypes as described below, suggesting that the phenotypes were not off target effects. As such, we only report the data from the NFIX KD1 construct here. Importantly, knockdown of *NFIX* did not result in any compensatory changes in the expression of *NFIA* or *NFIB* (Figure S3).

**Figure 5:**
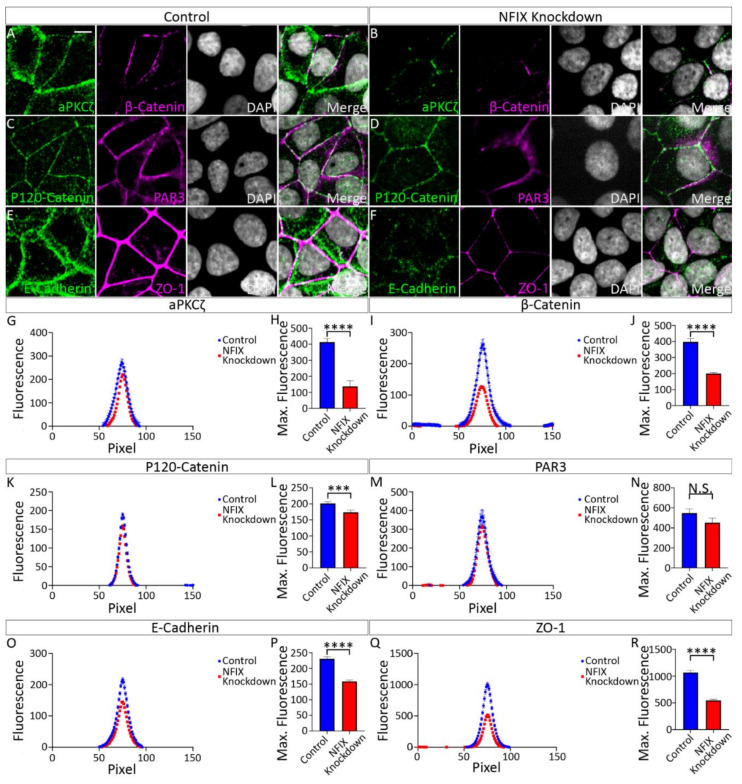
MCF7 cells treated with NFIX knockdown lentivirus demonstrate reduced localisation of adhesion proteins. (**A**,**B**) Images depicting MCF7 cells treated with scramble RNA lentivirus (scramble, **A**) or NFIX shRNA lentivirus (NFIX KD, **B**) and stained with antibodies against tight junction marker aPKCζ (green), adherens junction marker β-Catenin (purple) and nuclear marker DAPI (grey) displayed as separate panels or merged. (**C**,**D**) Images depicting NFIX KD (**C**) and scramble (**D**) cells stained with antibodies against adherens junction marker P120-Catenin (green), tight junction marker PAR3 (purple) and nuclear marker DAPI (grey) displayed as separate panels or merged (**E**,**F**) Images depicting NFIX KD (**E**) and scramble (**F**) cells stained with antibodies against adherens junction marker E-Cadherin (green), tight junction marker ZO-1 (purple) and nuclear marker DAPI (grey) displayed as separate panels or merged. (**G**,**I**,**K**,**M**,**O**,**Q**) Average fluorescent intensity across the cell junction was plotted for line scanning measurements of aPKCζ (**G**), β-Catenin (**I**), P120-Catenin (**K**), PAR3 (**M**), E-Cadherin (**O**) and ZO-1 (**Q**) for NFIX KD cells and controls. Tight junction proteins aPKCζ and ZO-1 demonstrated reduced fluorescent intensity peaks in NFIX KD cells compared to controls. Tight junction protein PAR3 demonstrated no changes between treatment conditions. Adherens junction proteins β-Catenin, P120-Catenin and E-Cadherin demonstrated reduced peak fluorescence in NFIX KD treated cells compared to controls. (**H**,**J**,**L**,**N**,**P**,**R**) Maximum fluorescent intensity across the junction of aPKCζ (**H**), β-Catenin (**J**), P120-Catenin (**L**), PAR3 (**N**), E-Cadherin (**P**) and ZO-1 (**R**) for NFIX KD cells and controls. Tight junction proteins aPKCζ and ZO-1 demonstrate significant reduction in the maximum fluorescence of NFIX KD cells compared to controls. Tight junction protein PAR3 demonstrates no significant difference between treatment conditions. Adherens junctions proteins β-Catenin, P120-Catenin and E-Cadherin demonstrate significantly reduced maximum fluorescent intensity in NFIX KD cells compared to controls. *** *p* < 0.001, **** *p* < 0.0001, N.S. (not significant), two-tailed *t*-test. Graphs depict mean ± s.e.m. from three samples per genotype with an n of 150 junctions. Scale bar (**A**) represents 10 µm.

MCF7 cells were treated with control or knockdown constructs and analysed 48 h later. IF was performed against key adherens/tight junction proteins, then line scanning was performed to investigate protein localisation. Consistent with our in vivo data, we observed significantly reduced average fluorescence intensity of aPKCζ, β-Catenin, P120-Catenin and ZO-1 in NFIX KD1 treated cells in comparison to controls (Figure 5). Likewise, we observed significantly reduced maximum fluorescence intensity readings of aPKCζ, β-Catenin, P120-Catenin and ZO-1 in NFIX KD1 treated cells in comparison to controls (Figure 5). In contrast, we did not see any significant differences in the distribution of the tight junction protein PAR3 although there may be a trend towards significance as average maximum fluorescence was decreased in NFIX KD cells but not significantly. Collectively, these data support the mouse model, and indicate that NFIX plays an important role in the establishment and/or maintenance of cell-cell contacts in epithelial cells.

### 3.3. Profiling of MCF7 Cell mRNA Transcription Reveals Downregulation of Adhesion Genes in NFIX KD Cells

We next examined whether changes in junctional localisation of epithelial adhesion proteins in NFIX KD cells compared to controls were the result of reduced transcription. We treated MCF7 cells with NFIX KD or control lentivirus and collected them as previously described. From NFIX KD and control cells we extracted mRNA using a miRNeasy Mini kit (QIAGEN Hilden, Germany) which was converted into cDNA using the “RT^2^ First Stand” kit (QIAGEN Hilden, Germany). Samples of cDNA from NFIX KD and control cells were then subjected to qPCR using “human extracellular matrix and adhesion molecules” and “human adherens junctions proteins” RT^2^ qPCR arrays. Genes that were found to be significantly upregulated or downregulated were also screened in silico for potential NFI binding sites using the predicted binding sites [44]. From this analysis, *CTNND1* (P120-Catenin) was found to be significantly downregulated in NFIX KD cells compared to controls. In contrast, *CHD1* (E-Cadherin) and *CTNNB1* (β-Catenin) had no significant difference between treatments (Table 3). Unexpectedly, *TJP1* (ZO-1) was found to be significantly upregulated in NFIX KD cells (Table 3). This suggests that reduced junctional localisation of β-Catenin, E-Cadherin and ZO-1 results from mechanisms other than reduced transcription.

In addition to *CDH1*, *CTNNB1*, *CTNND1* and *TJP1,* we also assayed for changes in the expression of other adherens junction genes and ECM genes via qPCR (Table 4 and Table S1). We observed downregulation of genes associated with adherens junction proteins in NFIX KD cells compared to controls including *ACTN4*, *CSNK2A2*, *CTNNA3*, *CTNND1*, *DSG1* and *DSG3* (Table 4). These genes encode for a range of adherens junction organising proteins, catenins, and desmosomal cadherins and their downregulation in NFIX KD cells may indicate abnormal adherens junction structure compared to controls which may impair epithelial cell-cell adhesion. In contrast, some adherens junction associated genes were upregulated in NFIX KD cells compared to controls including *ACTN3*, *CDSN*, *DSP*, *NME1*, *PKP2*, *TJP1* and *WASF1* (Table 4). We also found that ECM associated genes were downregulated in NFIX KD cells compared to controls including *CD44*, *COL6A1*, *COL14A1*, *ICAM1*, *ITGA3*, *ITGA4*, *ITGB1*, *LAMA2*, *LAMB3*, *LAMC1*, *MMP12*, *MMP14*, *SELL*, *SPP1*, *THBS2* and *TIMP3* (Table S2). These genes encode for proteins associated with a broad range of functions including ECM component proteins, ECM binding proteins and ECM remodelling proteins. In contrast *COL11A1*, and *TNC* were both found to be upregulated in NFIX KD cells compared to controls (Table S2).

### 3.4. NFIX Is Essential for Maintenance of Terminal Ependymal Cell Identity and Function in Adult Mice

#### 3.4.1. Tamoxifen Treatment of Adult *Nfix^iFOXJ1−GFP^* Mice Results in Decreased NFIX Expression in Ependymal Cells

**Figure 6:**
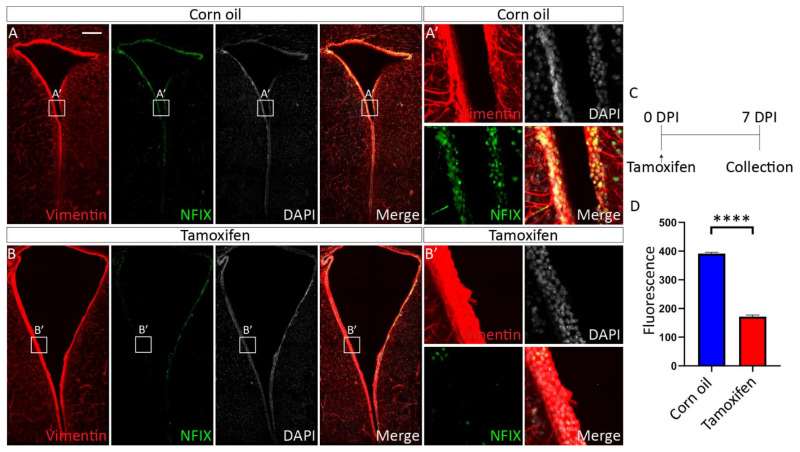
Tamoxifen treatment results in reduced NFIX expression in *Nfix^iFOXJ1−GFP^* mice. (**A**,**B**) Depth matched images of the lateral ventricles from adult corn oil (**A**) and tamoxifen (**B**) treated *Nfix^iFOXJ1−GFP^* mice stained with antibodies against ependymal marker Vimentin (red), transcription factor NFIX (green) and nuclear marker DAPI (grey) displayed separately and merged. (**A’**,**B’**) Regions depicted in boxes (**A’**,**B’**) are enlarged in panels (**A’**,**B’**). Tamoxifen treated *Nfix^iFOXJ1−GFP^* mice have visibly reduced expression of NIFX. (**C**) diagram of the tamoxifen treatment protocol including injections and tamoxifen dose. (**D**) Fluorescent intensity measurements of NFIX in adult *Nfix^iFOXJ1−GFP^* ependymal cells treated with tamoxifen compared to controls. Tamoxifen treated *Nfix^iFOXJ1−GFP^* mice demonstrate significantly reduced expression of NFIX compared to controls. **** *p* < 0.0001, two−tailed *t*-test. Graphs depict mean ± s.e.m. from three samples per genotype, 50 nuclei per sample. Scale bar (**A**) represents 200 µm.

**Figure 7:**
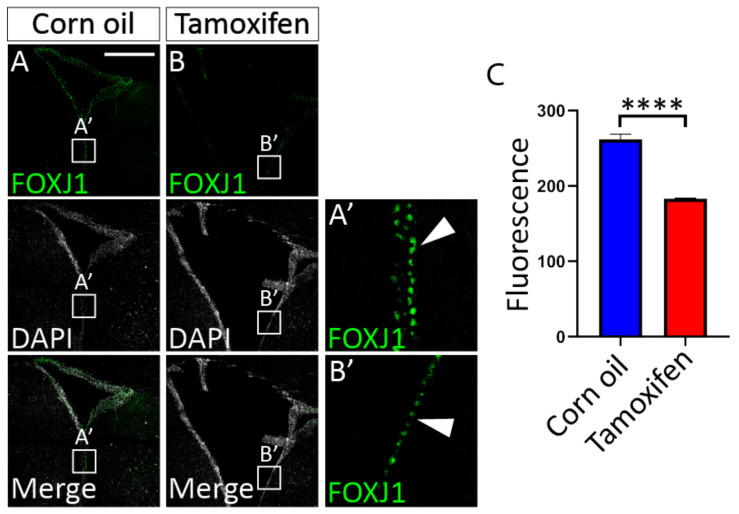
Loss of NFIX from adult ependymal cells resulted in reduced FOXJ1 expression. (**A**,**B**) Depth matched images of the lateral ventricles from adult corn oil (**A**) and tamoxifen (**B**) treated *Nfix^iFOXJ1−GFP^* mice stained with antibodies against ependymal marker FOXJ1 (green) and nuclear marker DAPI (grey) displayed separately and merged. (**A’**,**B’**) Regions depicted in boxes (**A’**,**B’**) are enlarged in panels (**A’**,**B’**). (**A’**,**B’**) Images depict FOXJ1 positive cells with a white arrowhead indicating a single FOXJ1^+^ cell. Tamoxifen treated *Nfix^iFOXJ1−GFP^* mice have visibly reduced expression of FOXJ1. (**C**) Fluorescent intensity measurements of FOXJ1 in adult *Nfix^iFOXJ1−GFP^* ependymal cells treated with tamoxifen compared to controls. Tamoxifen treated *Nfix^iFOXJ1−GFP^* mice demonstrate significantly reduced expression of NFIX compared to controls. **** *p* < 0.0001, two-tailed *t*-test. Graphs depict mean ± s.e.m. from three samples per genotype, 50 nuclei per sample. Scale bar (**A**) represents 300 µm.

**Figure 8:**
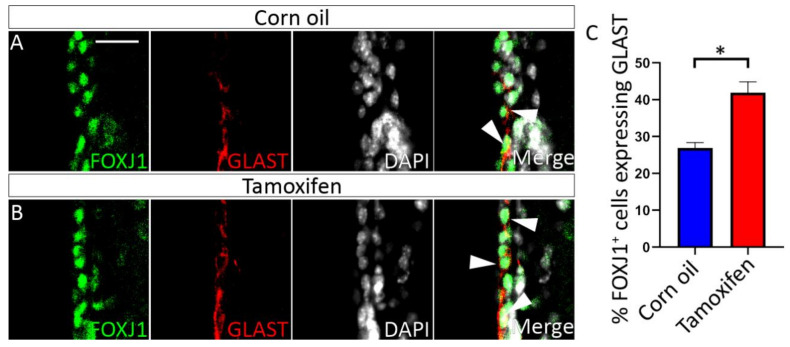
Some tamoxifen treated *Nfix^iFOXJ1−GFP^* FOXJ1^+^ ependymal cells express GLAST. (**A**,**B**) Lateral ventricle sections of corn oil (**A**) and tamoxifen (**B**) treated adult *Nfix^iFOXJ1−GFP^* ependyma marked with antibodies against ependymal marker FOXJ1 (green), radial glia marker GLAST (red) and nuclear marker DAPI (grey), displayed as separate images or merged. White arrowheads are used to mark FOXJ1^+^, GLAST^+^ cells. (**C**) The percentage of FOXJ1^+^ cells that also expressed GLAST is displayed for tamoxifen treated *Nfix^iFOXJ1−GFP^* ependymal cells and controls. The percentage of FOXJ1^+^ cells expressing GLAST is higher in tamoxifen treated *Nfix^iFOXJ1−GFP^* mice compared to controls. * *p* < 0.05 two−tailed *t*-test. Graphs depict mean ± s.e.m. from three samples per genotype. Scale bar (**A**) represents 25 µm.

NFIX is expressed in ependymal cells of the adult mouse brain [45]. Given that NFIX is essential for ependymal cell development, could NFIX play a role in the maintenance of ependymal cell function in adult cells? To test this, we developed an inducible NFIX knockout mouse model (*Nfix^iFOXJ1−GFP^* mice) which allowed the ablation of *Nfix* from ependymal cells following application of tamoxifen (Figure S1). Seven days post tamoxifen treatment, the expression of NFIX in lateral ventricle ependymal cells of *Nfix^iFOXJ1−GFP^* mice was significantly reduced in comparison to corn oil-treated control animals (Figure 6) as determined by reduced fluorescence of NFIX in tamoxifen treated mice compared to controls (Figure 6C). Consistent with the phenotype seen in ependymal cells in *Nfix KO* mice, we also saw reduced expression of FOXJ1 in ependymal cells of the lateral ventricles of tamoxifen treated *Nfix^iFOXJ1−GFP^* mice (Figure 7) as demonstrated by reduced fluorescence in FOXJ1 stains of tamoxifen *Nfix^iFOXJ1−GFP^* mice in comparison to controls (Figure 7C). The loss of FOXJ1 from mature ependymal cells has been associated with increased expression of GLAST, a developmental marker associated with neural progenitor cells and immature ependymal cells [46]. Interestingly, both corn oil and tamoxifen treated *Nfix^iFOXJ1−GFP^* mice demonstrated FOXJ1 and GLAST co-staining (Figure 8). FOXJ1 positive cells are not normally associated with GLAST expression in the lateral ventricles (Figure S4) however given that loss of FOXJ1 is associated with GLAST expression in ependymal cells [46], heterozygosity of FOXJ1 may have resulted in some FOXJ1 positive cells expressing GLAST in *Nfix^iFOXJ1−GFP^* mice.

Cell counts of GLAST^+^/FOXJ1^+^ cells demonstrated a significant increase in the number of double positive cells in tamoxifen treated *Nfix^iFOXJ1−GFP^* mice compared to controls (Figure 8C). These data suggest a role for NFIX in maintaining ependymal cell maturity within the adult brain.

**Figure 9:**
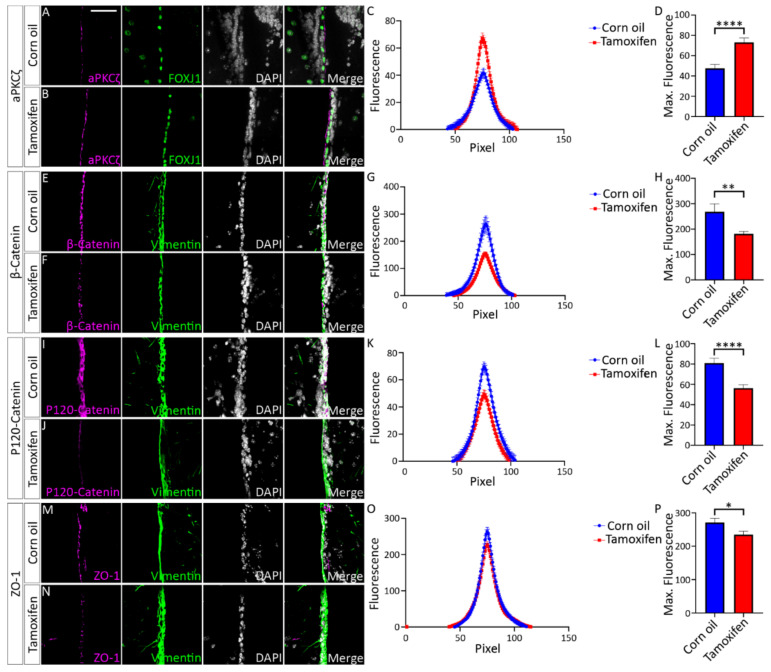
Tamoxifen treated *Nfix^iFOXJ1−GFP^* ependymal cells demonstrate reduced expression of adhesion proteins. (**A**,**B**) Lateral ventricle sections from *Nfix^iFOXJ1−GFP^* mice treated with corn oil (**A**) and tamoxifen (**B**) at 7 dpi labelled with aPKCζ (purple), ependymal cell marker FOXJ1 (green) and nuclear marker DAPI (grey), displayed separately and merged. (**C**) Average aPKCζ fluorescent intensity across the cell junction is displayed for tamoxifen treated *Nfix^iFOXJ1−GFP^* ependymal cells and controls. Average aPKCζ fluorescent intensity across the cell junction was increased at the peak in tamoxifen treated *Nfix^iFOXJ1−GFP^* ependymal cells compared to controls. (**D**) Maximum aPKCζ fluorescent intensity is displayed for tamoxifen treated *Nfix^iFOXJ1−GFP^* ependymal cells and controls. Maximum aPKCζ fluorescent intensity is significantly increased in tamoxifen treated *Nfix^iFOXJ1−GFP^* ependymal cells compared to controls. (**E**,**F**) Lateral ventricle sections from *Nfix^iFOXJ1−GFP^* mice treated with corn oil (**E**) and tamoxifen (**F**) at 7 dpi labelled with β-Catenin (purple), ependymal cell marker Vimentin (green) and nuclear marker DAPI (white), displayed separately and merged. (**G**) Average β-Catenin fluorescent intensity across the cell junction is displayed for tamoxifen treated *Nfix^iFOXJ1−GFP^* ependymal cells and controls. Peak β-Catenin fluorescent intensity is reduced in tamoxifen treated *Nfix^iFOXJ1−GFP^* mice compared to controls. (**H**) Maximum β-Catenin fluorescent intensity across the junction measured from tamoxifen treated *Nfix^iFOXJ1−GFP^* ependymal cells and controls. Maximum β-Catenin fluorescence was significantly reduced in tamoxifen treated *Nfix^iFOXJ1−GFP^* treated mice compared to controls. (**I**,**J**) Lateral ventricle sections from *Nfix^iFOXJ1−GFP^* mice treated with corn oil (**I**) and tamoxifen (**J**) at 7 dpi labelled with P120-Catenin (purple), ependymal cell marker Vimentin (green) and nuclear marker DAPI (white), displayed separately and merged. (**K**) Average P120-Catenin fluorescent intensity across the cell junction is displayed for tamoxifen treated *Nfix^iFOXJ1−GFP^* ependymal cells and controls. Peak P120-Catenin fluorescent intensity is reduced in tamoxifen treated *Nfix^iFOXJ1−GFP^* mice compared to controls. (**L**) Maximum P120-Catenin fluorescent intensity across the junction measured from tamoxifen treated *Nfix^iFOXJ1−GFP^* ependymal cells and controls. Maximum P120-Catenin fluorescence was significantly reduced in tamoxifen treated *Nfix^iFOXJ1−GFP^* treated mice compared to controls. (**M**,**N**) Lateral ventricle sections from *Nfix^iFOXJ1−GFP^* mice treated with corn oil (**M**) and tamoxifen (**N**) at 7 dpi labelled with ZO-1 (purple), ependymal cell marker Vimentin (green) and nuclear marker DAPI (white), displayed separately and merged. (**O**) Average ZO-1 fluorescent intensity across the cell junction is displayed for tamoxifen treated *Nfix^iFOXJ1−GFP^* ependymal cells and controls. Peak ZO-1 fluorescent intensity is reduced in tamoxifen treated *Nfix^iFOXJ1−GFP^* mice compared to controls. (**P**) Maximum ZO-1 fluorescent intensity across the junction measured from tamoxifen treated *Nfix^iFOXJ1−GFP^* ependymal cells and controls. Maximum ZO-1 fluorescence was significantly reduced in tamoxifen treated *Nfix^iFOXJ1−GFP^* treated mice compared to controls. * *p* < 0.05, ** *p* < 0.01, **** *p* < 0.0001, two-tailed *t*-test. Graphs depict mean ± s.e.m. from three samples per genotype with an n of 150 junctions. Scale bar (**A**) represents 50 µm.

Does the reduction in NFIX expression in the adult impact cell-cell adhesion? To analyse this, we next performed immunostaining against aPKCζ, β-Catenin, P120-Catenin and ZO-1 in ependymal cells marked with FOXJ1 or Vimentin in tamoxifen and corn oil treated adult *Nfix^iFOXJ1−GFP^* mice. From these stains we assessed protein localisation to the cell-cell junction using line scanning analysis as previously described. Consistent with the phenotype of postnatal *Nfix KO* mice, we revealed a decrease in the peak fluorescence of average fluorescence plots of β-Catenin, P120-Catenin and ZO-1 in tamoxifen treated mice compared to controls (Figure 9G–P). We also found significantly reduced maximum fluorescence in maximum fluorescence intensity plots of β-Catenin, P120-Catenin and ZO-1 in tamoxifen treated *Nfix^iFOXJ1−GFP^* mice compared to controls (Figure 9E–P). In contrast to previously observed results, aPKCζ was found to have increased peak fluorescence in average fluorescence intensity plots of tamoxifen treated *Nfix^iFOXJ1−GFP^* mice compared to controls (Figure 9C). Similarly, aPKCζ maximum fluorescence was found to be significantly increased in maximum fluorescence intensity plots of tamoxifen treated *Nfix^iFOXJ1−GFP^* mice compared to controls (Figure 9D).

#### 3.4.2. Loss of NFIX from Mature Ependymal Cells Leads to Ventricular Expansion

**Figure 10:**
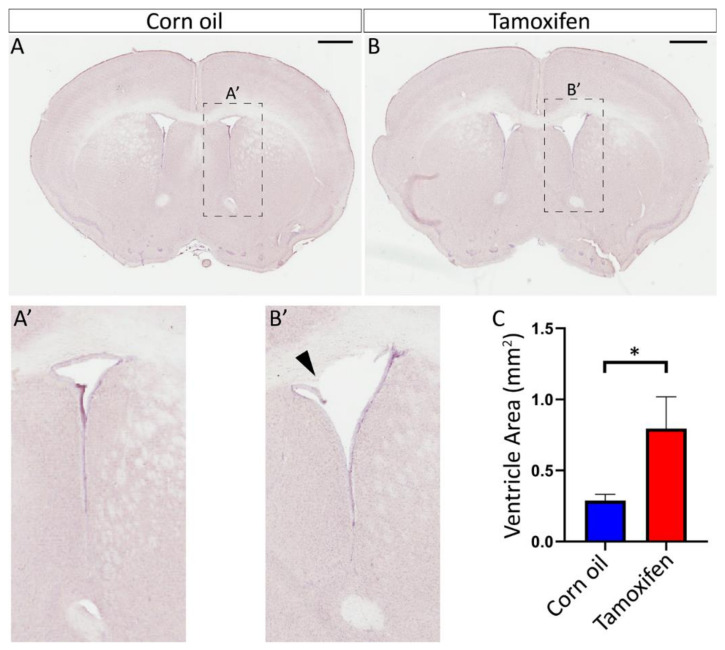
Tamoxifen treated *Nfix^Foxj1−GFP^* mice have enlarged ventricles. (**A**,**B**) Depth matched sections of lateral ventricle tissue from *Nfix^iFOXJ−GFP^* mice treated with corn oil (**A**) and tamoxifen (**B**). (**A’**,**B’**) Dashed boxes (**A’**,**B’**) are enlarged in panels (**A’**,**B’**). (**B’**) Sloughing of the ependyma occurs in the dorsal lateral ventricles of some tamoxifen treated *Nfix^iFOXJ−GFP^* mice (black arrowhead). (**C**) Measurements of ventricular area is displayed for tamoxifen treated *Nfix^iFOXJ1−GFP^* mice and controls. Ventricle area is significantly increased in tamoxifen treated *Nfix^iFOXJ1−GFP^* mice. * *p* < 0.05 two-tailed *t*-test. Graphs depict mean ± s.e.m. from seven samples per genotype. Scale bar (**A**,**B**) represents 1 mm.

The expansion of the lateral ventricles, leading to hydrocephaly, is a consistent phenotype in constitutively deleted *Nfix* mice [14,16]. Given that our adult inducible model presented with similar reductions of FOXJ1 expression (Figure 7) and aberrant adherens and tight junction protein localisation in comparison to the *Nfix KO* line (Figure 9), we postulated that our inducible model would also exhibit enlarged lateral ventricles at 7 days post tamoxifen treatment. To determine if this was the case, we cut 50 µm thick coronal sections and stained them with haematoxylin. We then measured the area of the lateral ventricles in tamoxifen treated mice in comparison to controls (Figure 10). Consistent with our hypothesis, the lateral ventricles of tamoxifen-treated *Nfix^iFOXJ1−GFP^* mice were significantly larger than the controls (Figure 10C), despite the relatively short duration of the experiment. Furthermore, we consistently observed areas within the dorsal region of the lateral ventricles of tamoxifen treated animals where the ependymal layer was sloughing off (Figure 10B’), suggestive of aberrant cell-cell adhesion in these mice.

## 4. Discussion

### 4.1. NFIX Drives Acquisition and Maintenance of Ependymal Cell Identity Traits

Previous studies examining the role of NFIX in ependymal cells have demonstrated a role for NFIX in ependymal development [14] and ependymal cell function [16]. In this study we have expanded upon these finding by demonstrating a role for NFIX in driving acquisition and maintenance of ependymal cell fate. We demonstrate that at P5, *Nfix^−/−^* ependymal cells have prolonged expression of immature ependymal cell traits including the radial glia marker PAX6 and display a rounded cell morphology. These traits indicate that maturation is delayed in *Nfix^−/−^* ependymal cells and shows that NFIX is essential for the timing of ependymal maturation. We also show that removal of NFIX from adult ependymal cells is associated with loss of ependymal cell function indicated by ventricular expansion and sloughing of the ependyma from the dorsal ventricles of tamoxifen-treated *Nfix^iFOXJ1−GFP^* mice. Furthermore, we found increased expression of GLAST in tamoxifen treated *Nfix^iFOXJ1−GFP^* ependymal cells compared to controls, indicating ependymal immaturity, and showing that NFIX is involved in the maintenance of ependymal cell identity. Together these results demonstrate that NFIX plays a role in driving ependymal cell fate both developmentally and in the adult.

One mechanism through which NFIX may modulate both the acquisition and maintenance of ependymal cell fate is through chromatin modification. Previous studies have demonstrated a role for NFIX in the regulation of chromatin [47,48,49,50,51,52,53]. Within hair follicle (HF) bulge skin stem cells (bulge-SCs) NFIB and NFIX maintain chromatin access to enable H3K4me1 and H3K27ac modified nucleosomes for modulation of super-enhancers [51]. Loss of NFIB and NFIX in bulge-SCs lead to closure of bulge-SC specific super-enhancer regions resulting in gradual loss of HFs [51]. Although bulge-SCs are not a terminally differentiated population like ependymal cells, their distinctive cell fate from epidermal skin cells is maintained by NFIB and NFIX as they form a barrier to plasticity. Could NFIX also drive the acquisition and maintenance of ependymal cell identity traits through chromatin modification? Future studies could potentially answer this by using single cell assay for transposase-accessible chromatin with high throughput sequencing (scATAC-seq) to investigate the role of NFIX in chromatin dynamics during ependymal development. These could be paired with a combination of chromatin immunoprecipitation sequencing (ChIP-seq) and single cell RNA sequencing (scRNA-seq) to examine how NFIX regulates the expression of transcription factors essential for ependymal development.

### 4.2. Expression of NFIX Is Essential for Cell Adhesion in Ependymal Cells

Alongside driving ependymal cell fate, we also identified a critical role for NFIX in the organisation of ependymal cell adhesion both developmentally and in the adult. We demonstrated that both P5 *Nfix^−/−^* and adult tamoxifen treated *Nfix^iFOXJ1−GFP^* ependymal cells exhibited reduced junctional localisation of key adhesion proteins β-Catenin, P120-Catenin and ZO-1 compared to controls. Furthermore, TEM of P5 mice demonstrates that the junctions of P5 *Nfix^−/−^* mice are structurally altered compared to controls. In the context of ependymal shedding observed in both *Nfix^−/−^* and tamoxifen treated *Nfix^iFOXJ1−GFP^* lateral ventricles, these findings indicate an adhesion deficit in NFIX-deficient ependymal cells. While this is a novel role for NFIX in the context of ependymal cells, NFIX has previously been shown to regulate adhesion in hematopoietic stem and progenitor cells (HSPCs) [54,55]. Mouse bone marrow transplants of NFIX negative HSPCs fail to engraft in recipient mice and gene expression profiling revealed significantly decreased expression of adhesion molecules including integrins and β-Catenin [54,55]. Perhaps more relevantly, NFIB has also been implicated in regulating the adhesion of epithelial cancers including small cell lung cancer (SCLC) [56,57,58]. In these cancers NFIB is thought to modulate chromatin structures to induce a stable epithelial-to-mesenchymal cell fate [56] which is associated with the decreased expression of epithelial cell adhesion markers such as E-Cadherin [58]. The decreased junctional localisation of E-Cadherin in NFIX KD MCF7 cells compared to controls suggests that NFIX may work through a similar mechanism.

Interestingly, qPCR of *CDH1* (E-Cadherin), *CTNNB1* (β-Catenin) and *TJP1* (ZO-1) demonstrated no difference between NFIX KD MCF7 cells and controls and indicates that abnormal junctional localisation is not due to decreased transcription. One mechanism that may explain this result could be increased internalisation of cadherin-catenin complexes via endocytosis. In support of this, *CTNND1* (P120-Catenin) was found to be decreased via qPCR and P120-Catenin demonstrated reduced junctional localisation in NFIX KD cells. P120-Catenin is a surface stabiliser of cadherin-catenin complexes as it binds to the endocytosis domain of cadherins preventing its interaction with endocytosis machinery [43,59,60,61]. Future research could examine this possibility by monitoring the internalisation of surface β-Catenin of ependymal cells labelled with a fluorescence antibody using live cell imaging.

Alongside changes in adherens junction proteins, qPCR of NFIX KD and control MCF7 cells also demonstrated significant decreased expression of genes associated with ECM proteins. Broadly, three groups of ECM associated genes were found to be downregulated in NFIX KD cells namely ECM component encoding genes, ECM remodelling and inhibitor proteins encoding genes, and ECM binding protein encoding genes. Of these categories, the downregulation of ECM binding protein encoding genes may lead to reduced ECM binding affinity in epithelial cells with reduced NFIX expression. Integrin subunit encoding genes *ITGA3*, *ITGA4* and *ITGB1* were downregulated in NFIX KD cells compared to controls via qPCR. ITGA3 combines with ITGB1 to form an integrin receptor that interacts with many members of the laminin family [62,63] while ITGA4 associates with ITGB1 to form an integrin associated with fibronectin or VCAM binding [63,64]. As such, downregulation of *ITGA3* and *ITGB1* may directly lead to decreased binding affinity for laminin and thereby reduced cell-to-ECM adhesion. Similarly, downregulation of *ITGA4* and *ITGB1* may directly lead to decreased binding affinity for fibronectin in NFIX KD cells and result in reduced cell-to-ECM adhesion. Downregulation of *ITGA3*, *ITGA4* and *ITGB1* in *Nfix^−/−^* and tamoxifen treated *Nfix^iFOXJ1−GFP^* ependymal cells may contribute to the sloughing phenotype observed in the dorsal lateral ventricles as the ependyma layer may have reduced binding affinity for the ECM of the ventricular wall. Future studies could examine the impact of reduced *ITGA3*, *ITGA4* and *ITGB1* on epithelial cell-to-ECM adhesion in NFIX KD cells compared to controls using plating assays for laminin and fibronectin.

### 4.3. Altered Cilia Structure in P5 Nfix^−/−^ Mice May Also Contribute to Hydrocephalus

Alongside changes to ependymal adhesion, this study also demonstrated altered cilia structure in P5 *Nfix^−/−^* ependymal cells under TEM. We observed the presence of additional microtubules in *Nfix^−/−^* ependymal cilia with some cilia displaying sporadic sets of triplets around the outside and sometimes 3 or 4 microtubules in the centre. Motile cilia are normally defined by a 9 + 2 pattern with 9 sets of doublets around the outside and 2 microtubules in the centre however some alternate forms exist [65]. Of note is the arrangement of 9 + 4 that has also been demonstrated by TEM in the rabbit notochordal plate which appears to be capable of rotational beating [66]. The authors of this paper mention that 9 + 4 cilia are expressed in the mouse posterior notochord as well [66] however no other published papers mention this finding. It is possible that a proportion of the cilia of *Nfix^−/−^* ependymal cells may still be motile although it is unclear if these cilia would be capable of coordinated beating, the disruption of which could contribute to *Nfix^−/−^* mouse hydrocephalus. Perhaps future studies could perform functional assays using ex−vivo slices of *Nifx^−/−^* mice by placing fluorescent beads on the ventricular surface and tracking their movement using live fluorescence microscopy.

Interestingly, rabbit notochordal 9 + 4 cilia are transient and are lost during development [66]. The authors posit that the transitory appearance of 9 + 4 cilia may be involved in left-right axis organisation [66]. As FOXJ1 is a known regulator of left-right axis formation in mice [24], could the appearance of additional microtubules have a relation to decreased FOXJ1 expression in *Nfix^−/−^* ependymal cells? At this stage it remains unclear but remains an exciting avenue for future research.

While ependymal cilia are disrupted in constitutive *Nfix^−/−^* mice it is unclear if this is the case in adult ependymal cells following loss of NFIX. Although there is turnover of cilia component proteins in adult cells [67] the rate of this turnover is unclear. Furthermore, as we did not visualise the structure of cilia in tamoxifen treated adult *Nfix^iFOXJ1−GFP^* ependymal cells it is unknown if these cells acquire additional microtubules. Interestingly, FOXJ1 is required for the maintenance of multicilia in mature ependymal cells [46]. Given that tamoxifen treated *Nfix^iFOXJ1−GFP^* ependymal cells have reduced FOXJ1 expression, could ependymal cilia number be reduced in tamoxifen treated *Nfix^iFOXJ1−GFP^* mice? Future studies could use TEM to further study cilia structure in *Nfix^iFOXJ1−GFP^* mice.

### 4.4. Mutations in NFIX Are Associated with Malan Syndrome in Humans

This study has demonstrated a role of NFIX in driving ependymal cell terminal identity, including the development of normal cilia and cell junction structures. These findings are highly relevant for understanding ependymal cell fate in patients with NFIX-related developmental disorders including Malan syndrome. Malan syndrome is characterised as an overgrowth disorder presenting unusual facial phenotypes, intellectual disability and behavioural abnormalities [68,69], and is a result of *NFIX* haploinsufficiency [70]. Although not specifically associated with hydrocephalus, some Malan syndrome patients present non-specific enlargement of the ventricles [68]. As demonstrated by MCF7 cell culture, NFIX is vital for the maintenance of normal epithelial adhesion, and 60–80% reduction in NFIX is sufficient to induce significant changes in the junctional localisation of adhesion proteins. Therefore, it is plausible that Malan syndrome patients may have abnormal ependymal cell junctions and cilia, which may contribute to the expansion of the ventricles, even with heterozygous NFIX expression. Future studies could more directly model the effects of Malan syndrome on ependymal cell morphology using ependymal cells derived from induced pluripotent stem cells (IPSCs). One limitation that currently prevents the use of IPSCs for ependymal cell modelling is the lack of protocols for differentiating ependymal cells from IPSCs. While there is some evidence to suggest that ependymal-like cells may be induced in vitro, these cells more closely resemble endothelial blood brain barrier cells than ependymal cells [71]. As such, a new protocol would have to be established before attempting to use iPSCs to model ependymal cells in Malan syndrome. Despite this, such research is vital for understanding the Malan syndrome phenotype and may potentially lead to potential treatments in the future.

## Figures and Tables

**Table 1:** Antibodies used in immunofluorescence histochemistry of fixed *Nfix KO* and *Nfix^iFOXJ1-GFP^* brain tissue sections. Table list the name, species, manufacturer and the concentration of use for each antibody. Antibody concentrations diluted in donkey block.

Antibody Name/Target	Catalogue Number	Host Species	Manufacturer	Concentration
**Primary Antibodies**				
aPKCζ	Sc-216	Rabbit	Santa Cruz	1:300
β-Catenin	05-655	Mouse	Millipore	1:300
P120-Catenin	33-9600	Rabbit	Abcam	1:300
ZO-1	40-2300	Mouse	Invitrogen	1:300
NFIX	SAB1401263	Mouse	Sigma-Aldrich	1:400
FOXJ1	MA5-31419	Mouse	eBioscience	1:500
Vimentin	ab92547	Rabbit	Abcam	1:500
PAX6	ab78545	Mouse	Abcam	1:500
**Secondary Antibodies**				
Donkey α Rabbit Cy3	AB_2307443	Donkey	Jackson ImmunoResearch	1:300
Donkey α Mouse 647	AB_2340863	Donkey	Jackson ImmunoResearch	1:300
DAPI		-	Thermofisher	1:1000

**Table 2:** Antibodies used for immunofluorescence histochemistry conducted on fixed MCF7 cells. Table list the name, species, manufacturer and the concentration of use for each antibody. Antibody concentrations diluted in donkey block.

Antibody Name/Target	Catalogue Number	Host Species	Manufacturer	Concentration
	**Primary Antibodies**			
PAX6	ab78545	Rabbit	Abcam	1:500
aPKCζ	Sc-216	Rabbit	Santa Cruz	1:300
β-Catenin	05-655	Mouse	Millipore	1:300
E-Cadherin	24E10	Rabbit	Cell Signalling	1:300
PAR3	07-033	Rabbit	Millipore	1:300
P120-Catenin	33-9600	Rabbit	Abcam	1:300
ZO-1	40-2300	Mouse	Invitrogen	1:300
	**Secondary Antibodies**			
Donkey α Rabbit Cy3	AB_2307443	Donkey	Jackson ImmunoResearch	1:300
Donkey α Mouse 647	AB_2340863	Donkey	Jackson ImmunoResearch	1:300
DAPI		-	Thermofisher	1:1000

**Table 3:** Results of qPCR conducted against genes encoding for junction proteins which showed significant differences during line scanning analysis. *CDH1* and *CTNNB1* were found to have no significant differences in expression between treatment groups via *t*-test. *CTNND1* was found to have significantly downregulated expression via *t*-test. *TJP1* was found to have significantly upregulated expression via *t*-test. As assayed genes were screened against a database of predicted NFI binding sites in silico and the position of predicted binding sites reported.

				Average Raw C_T_		
Gene	Protein	Fold Change	Student *t*-Test *p*-Value	Scramble	Knockdown	Predicted NFI Binding Site
*CDH1*	E-Cadherin	1.08	0.449571	1.96	1.84	−1806, −51
*CTNNB1*	Β-Catenin	0.94	0.608607	5.31	5.4	−2418, −1040, −862, −335,
*CTNND1*	P120-Catenin	0.72	0.040615	3.99	4.47	−268
*TJP1*	ZO-1	1.13	0.019167	4.05	3.87	−628, −341

**Table 4:** Differential gene expression observed in *NFIX* knockdown lentivirus treated MCF7 cells as compared to scramble lentivirus treated cells as compared via a “human adherens junctions” and a “human extracellular matrix and adhesion molecules RT^2^ qPCR profiler array. Genes with a Fold change > 2 (red) or < 0.5 (blue) were considered to have significantly upregulated or downregulated expression respectively. Similarly, genes which were found to have a *p*-value < 0.05 via two-tailed students *t*-test were also considered to be significantly upregulated or downregulated respectively. Significant values were calculated using a cycle threshold of 0.095, similarly samples were checked for genomic contamination and mRNA integrity using in array controls. Samples were found to have good quality mRNA (CTPPC = 20 ± 2) and had a low chance of genomic contamination (CTHGDC ≥ 33). An n = 3 *NFIX* shRNA lentivirus MCF7 cells and a scramble RNA lentivirus MCF7 cells.

			Average Raw C_T_		
Gene	Fold Change	Student *t*-Test *p*-Value	Scramble	Knockdown	Predicted NFI Binding Site
*TNC*	3.36	0.37651	1.60E-01	9.50E-02	−98, −288, −2010
*ACTN3*	2.28	0.96452	1.00E−04	4.50E−05	−881, −1407, −1565, −1796, −476, −605, −704
*COL11A1*	2.14	0.20736	6.80E−06	1.50E−05	−2190, −1940, −888, −829
*CDSN*	1.4	0.04696	3.20E−01	3.80E−01	−1679, −1029, −248, +174
*NME1*	1.31	0.0055	1.60E−03	1.20E−03	−
*PKP2*	1.19	0.04353	5.20E−03	6.70E−03	−
*DSP*	1.16	0.02935	2.80E−06	6.00E−06	−
*WASF1*	1.16	0.0032	4.50E−02	6.30E−02	−
*TJP1*	1.13	0.01917	9.70E−06	3.00E−05	−341, −628
*ACTN4*	0.83	0.02028	3.00E−06	6.20E−06	−206, −1756, −2120
*ITGB1*	0.82	0.0338	3.40E−04	9.80E−05	−2797, −2933, −2671, −2807
*LAMC1*	0.77	0.03058	8.90E−03	5.20E−03	−176, −205, −629, −2467, −2913
*CSNK2A2*	0.77	0.0431	1.20E−01	1.10E−01	−169, −1669
*CTNND1*	0.72	0.04062	1.40E−01	1.10E−01	−268
*ITGA3*	0.71	0.02321	7.10E−03	3.80E−03	−319, −619, −884, −1044, −1051, −1362, −2203, −2329, −2351, −2170, −2874, −2881, −2995
*SELL*	0.6	0.01999	6.20E−02	4.40E−02	−308, −1285
*COL6A1*	0.59	0.00512	6.90E−05	1.10E−05	−1184, −1485, −2312
*CD44*	0.58	0.01301	2.40E−01	2.00E−01	−
*ICAM1*	0.54	0.00981	1.50E−04	7.10E−05	−2849, −1716, −719
*LAMA2*	0.49	0.38715	6.30E−02	2.60E−02	−1489, −463, −1320, −2657
*SPP1*	0.48	0.25159	9.70E−02	7.50E−02	−1929, −3010
*DSG3*	0.48	0.37356	1.70E−02	1.40E−02	−741
*CTNNA3*	0.46	0.07407	6.80E−02	6.00E−02	−297, −1970, −1935, −2262, −5
*TIMP3*	0.44	0.02463	2.00E−03	6.60E−04	−2954
*LAMB3*	0.41	0.13912	3.60E−04	1.40E−04	−1960, −1821, −549, −473, −214, +189
*MMP14*	0.41	0.05487	6.50E−03	3.90E−03	−2947, −2626, −1745, −976, −243
*MMP12*	0.34	0.5996	1.10E−05	5.10E−06	−1065
*DSG1*	0.32	0.23532	8.70E−03	7.50E−03	−1833, −1805, −1420, −1489
*COL14A1*	0.29	0.3944	1.40E−04	2.20E−05	−326, −230, −173,
*ITGA4*	0.16	0.03347	1.30E−02	5.90E−03	−2912, −78, +4, +10
*THBS2*	0.15	0.00248	1.50E−05	5.10E−05	−2354

## Data Availability

Available on request to the corresponding author.

## Supplementary Materials

The following supporting information can be downloaded at: https://www.mdpi.com/article/10.3390/cells11152377/s1, Figure S1: Generation and tamoxifen treatment of the NfixiFOXJ1−GFP mouse line; Figure S2: Line scanning analysis protocol, Figure S3: MCF7 cells treated with NFIX knockdown lentivirus demonstrate reduced NFIX via qPCR, Figure S4: Tamoxifen treated NfixiFOXJ1−GFP mice demonstrate FOXJ1 and GLAST co-staining in contrast to wild type controls, Table S1: All genes screened via “human adherens junction genes” RT2 qPCR array, Table S2: All genes screened via “human extracellular matrix and adhesion molecules” RT2 qPCR array.
