# Supplementary material for: Hydrocephalus in Nfix−/− Mice Is Underpinned by Changes in Ependymal Cell Physiology
Source: Cells. 2022 Aug 2;11(15):2377. doi: 10.3390/cells11152377 (PMC9368351; doi:10.3390/cells11152377)
Supplement: Supplementary file 1 [file cells-11-02377-s001.zip › cells-1838354-supplementary.pdf]

cells

MDPI

Research Article

Hydrocephalus in *Nfix*$^{-/-}$ mice is underpinned by changes in ependymal cell physiology

[1]Danyon Harkins, [1]Tracey J. Harvey, [1]Cooper Atterton, [1]Ingrid Miller, [1]Laura Currey, [1]Sabrina Oishi, [1]Maria Kasherman, [1]Raul Ayala Davila, [2]Lucy Harris, [2]Kathryn Green, Hannah Piper, [2, 3]Robert G. Parton, [4]Helen M. Cooper, [1]Stefan Thor, [1, 4]Michael Piper

1 School of Biomedical Sciences, The University of Queensland, Brisbane 4072, Australia.
2 Centre for Microscopy and Microanalysis, The University of Queensland, Brisbane 4072, Australia.
3 Institute for Molecular Biosciences, The University of Queensland, Brisbane 4072, Australia.
4 Queensland Brain Institute, The University of Queensland, Brisbane 4072, Australia.
* Correspondence: m.piper@uq.edu.au

Citation: Harkins, D.; Harvey, T.J.; Atterton, C.; Miller, I.; Currey, L.; Oishi, S.; Kasherman, M.; Davila, R.A.; Harris, L.; Green, K.; et al. Hydrocephalus in Nfix−/− Mice Is Underpinned by Changes in Ependymal Cell Physiology. Cells 2022, 11, 2377. https://doi.org/10.3390/cells11152377

Academic Editor: David A. Jans and Jeremy M. Crook

Received: 13 July 2022
Accepted: 29 July 2022
Published: 2 August 2022

Publisher's Note: MDPI stays neu-tral with regard to jurisdictional claims in published maps and institu-tional affiliations.

Copyright: © 2022 by the authors. Submitted for possible open access publication under the terms and conditions of the Creative Commons Attribution (CC BY) license (https://creativecommons.org/licenses/by/4.0/).

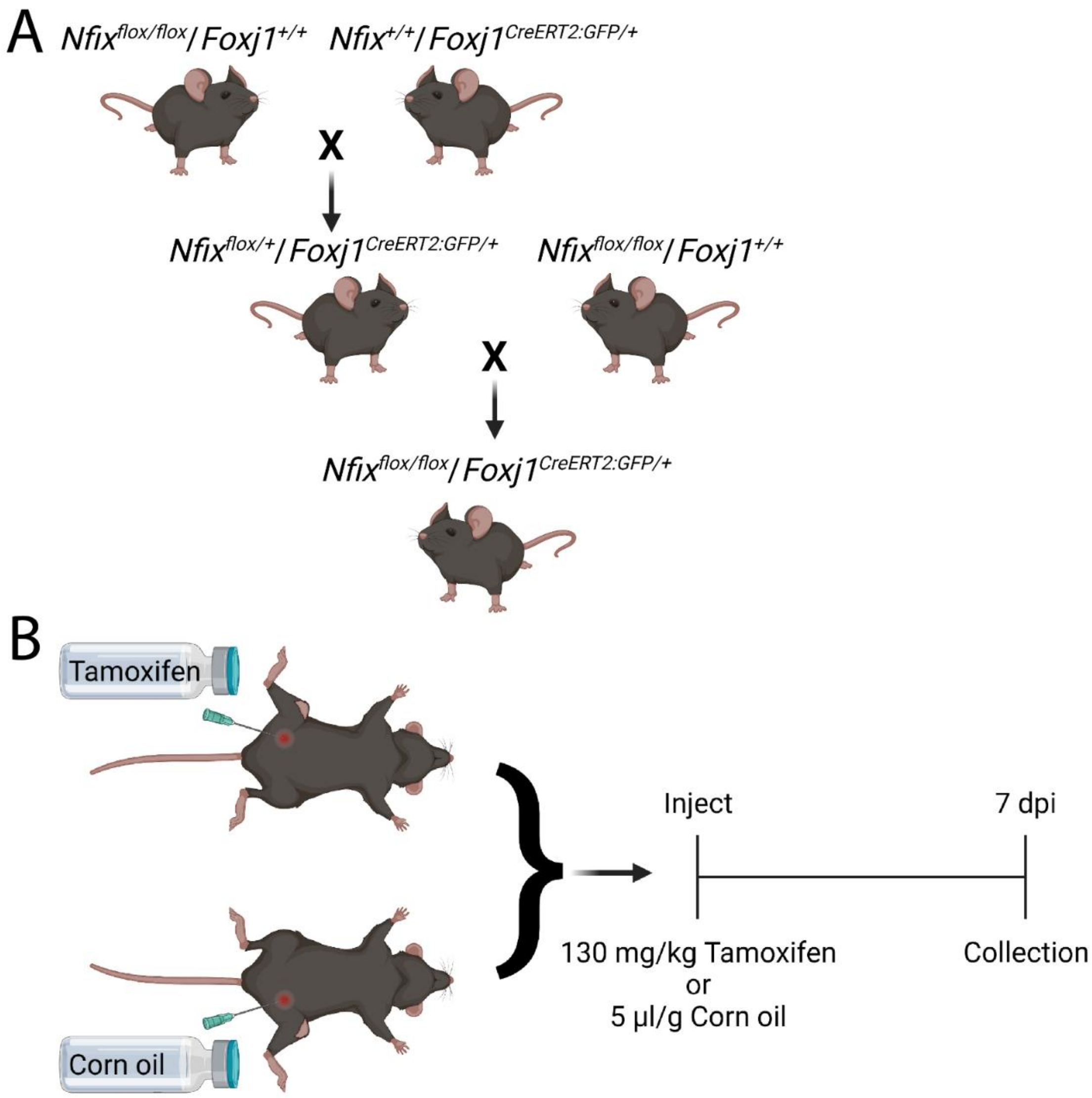

Figure S1. Generation and tamoxifen treatment of the *Nfix*$^{iFOXJ1\text{-}GFP}$ mouse line. (**A**) *Nfix*$^{+/+}$;*Foxj1*$^{CreERt2\text{-}GFP/+}$ mice were obtained and crossed with *Nfix*$^{flox/flox}$;*Foxj1*$^{+/+}$ mice. From the resulting progeny *Nfix*$^{flox/+}$;*Foxj1*$^{CreERt2\text{-}GFP/+}$ mice were selected and crossed to *Nfix*$^{flox/flox}$;*Foxj1*$^{+/+}$ mice. The crossing of these mice produced *Nfix*$^{flox/flox}$;*Foxj1*$^{CreERt2\text{-}GFP/+}$ mice among other genotypes. These *Nfix*$^{flox/flox}$;*Foxj1*$^{CreERt2\text{-}GFP/+}$ mice were further crossed with *Nfix*$^{flox/flox}$;*Foxj1*$^{+/+}$ mice to maintain the colony or were otherwise used as experimental animals. (**B**) *Nfix*$^{iFOXJ1\text{-}GFP}$ mice were treated with one intraperitoneal injection of 130 mg/kg tamoxifen dissolved in corn oil (experimental) or equivalent volume corn oil at 5 µl/g (control). Mice were monitored twice daily for signs of stress, changes in body weight or abnormal behaviour. Mice were collected for transcardial perfusion at 7 days post injection (dpi). Figure created with BioRender.com.

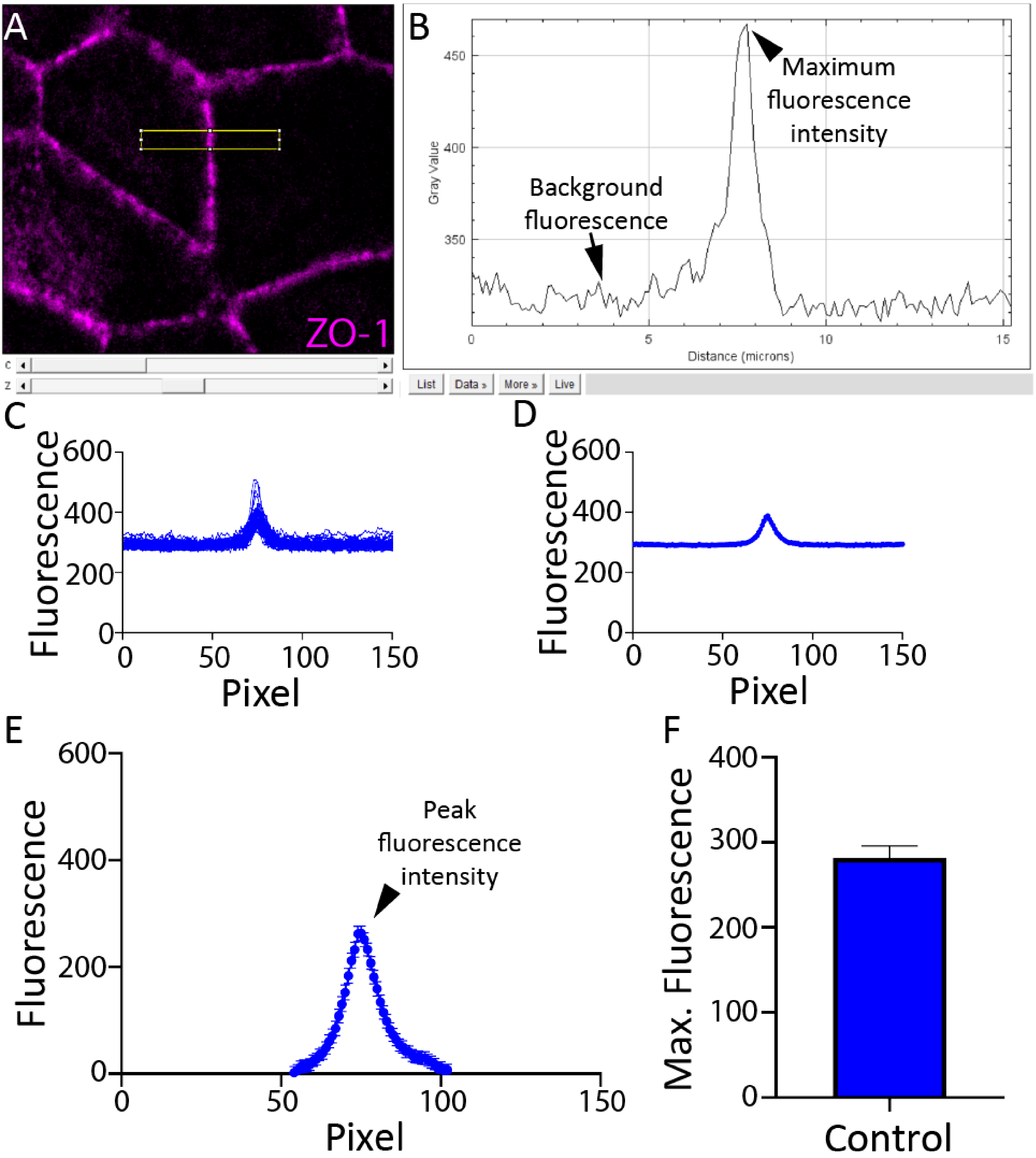

Figure S2. Line scanning analysis protocol. (**A**) Images of fluorescence stains against adhesion markers are opened in Fiji (ImageJ). Boxes 150 pixels by 20 pixels are drawn perpendicular to and centred on the cell junction (yellow). (**B**) Using the plot tool, average immunofluorescence is plotted across the cell junction. Black arrow depicts background fluorescence, black arrowhead depicts peak fluorescence. (**C**) Measurements are repeated 50 times per section along different cell junctions. (**D**) Average measurement is taken for each section. (**E**) A background correction is performed by subtracting the median fluorescent intensity from each point. (**F**) The maximum value from each measurement (maximum fluorescence) are collected and plotted separately. Graph depicts mean ± s.e.m.

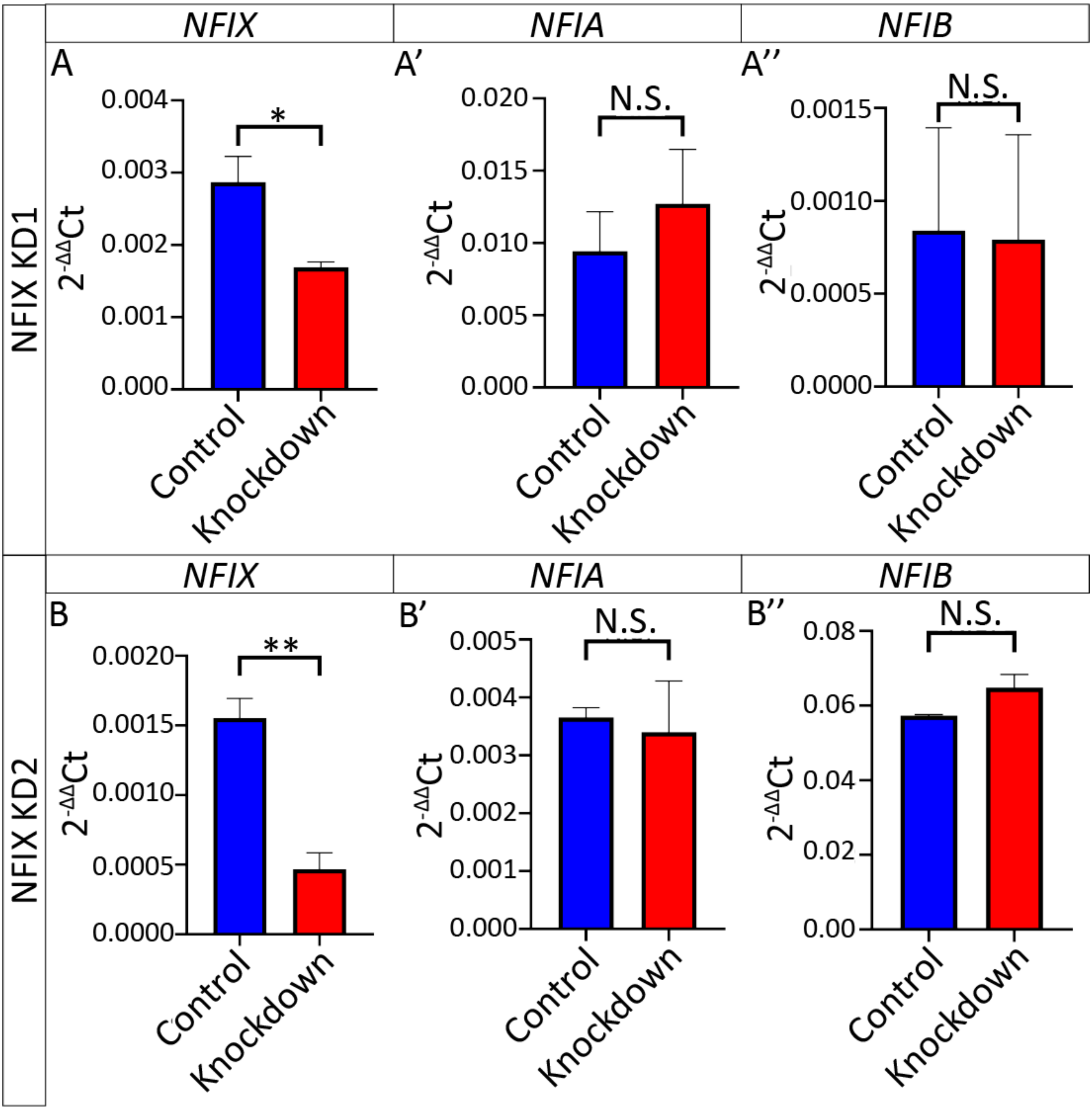

Figure S3. MCF7 cells treated with NFIX knockdown lentivirus demonstrate reduced *NFIX* via qPCR. (**A**,**B**) Panels depict $2^{-\Delta\Delta Ct}$ for *NFIX*, *NFIA* and *NFIB* normalised to housekeeping gene *GAPDH* for MCF7 cells treated with two different NFIX knockdown lentivirus constructs (NFIX KD1, **A**) (NFIX KD2, **B**) compared to scramble controls. *NFIX* expression in MCF7 cells treated with construct NFIX KD1 (**A**) and NFIX KD2 (**B**) demonstrate significant downregulation compared to controls. (**A′**,**B′**) No significant difference was observed in *NFIA* expression between treatment groups. (**A′′**,**B′′**) No significant difference in *NFIB* expression was observed between treatment groups. * $p<0.05$, ** $p<0.01$, two-tailed t-test. Graphs depict mean ± s.e.m. from three samples per genotype.

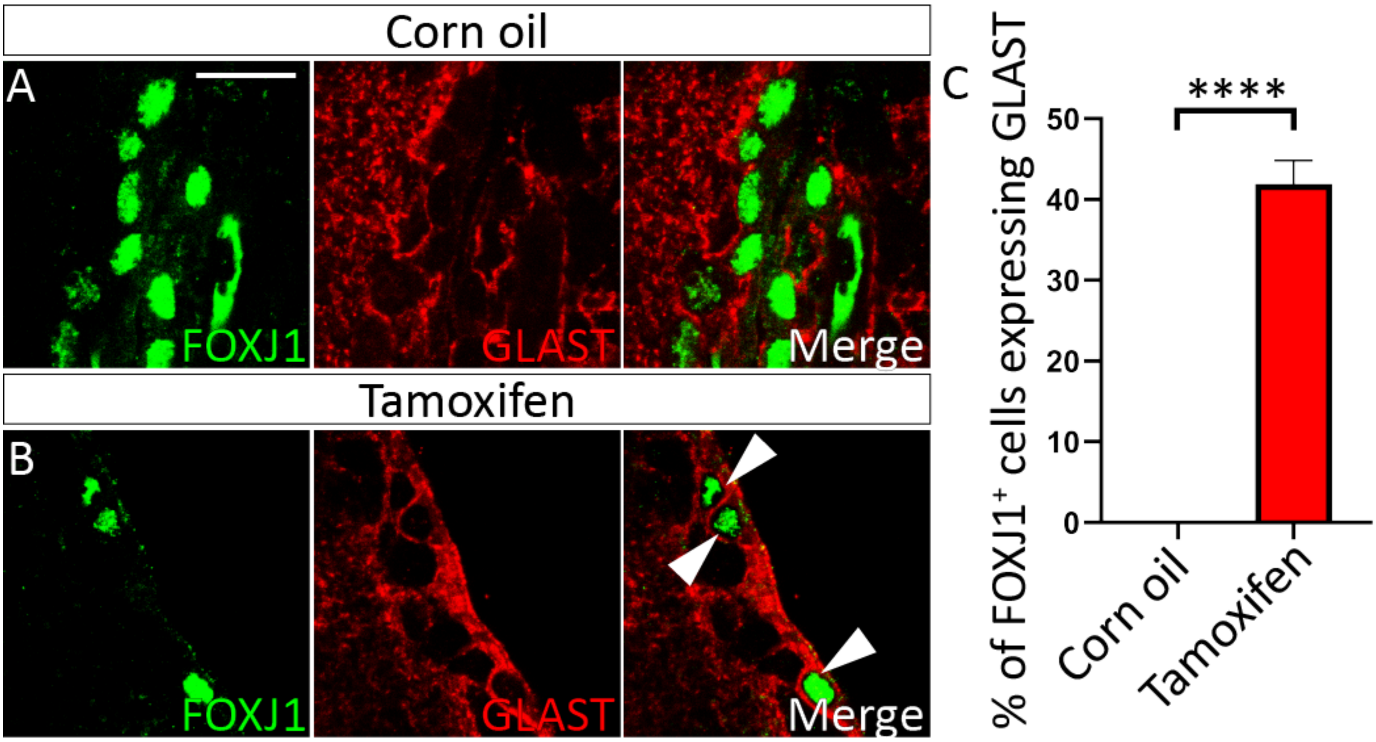

Figure S4. Tamoxifen treated *Nfix*$^{iFOXJ1\text{-}GFP}$ mice demonstrate FOXJ1 and GLAST co-staining in contrast to wild type controls. (**A**,**B**) Lateral ventricle sections of wild type (**A**) and tamoxifen treated adult *Nfix*$^{iFOXJ1\text{-}GFP}$ (**B**) ependyma marked with antibodies against ependymal marker FOXJ1 (green), radial glia marker GLAST (red) and nuclear marker DAPI (grey), displayed as separate images or merged. White arrowheads are used to mark FOXJ1$^+$, GLAST$^+$ cells. (**C**) The percentage of FOXJ1$^+$ cells that also expressed GLAST is displayed for tamoxifen treated *Nfix*$^{iFOXJ1\text{-}GFP}$ ependymal cells and controls. The percentage of FOXJ1+ cells expressing GLAST is higher in tamoxifen treated Nfixi-FOXJ1-GFP mice compared to controls. **** *p*<0.0001 two-tailed t-test. Graphs depict mean ± s.e.m. from three samples per genotype. Scale bar (**A**) represents 25 µm..

Table S1. All genes screened via "human adherens junction genes" RT² qPCR array. $2^{-\Delta\Delta Ct}$ scores were calculated relative to *GAPDH* as a normalising housekeeping gene. Genes with a fold change > 2 or < 0.5 have had their fold change marked in bold. Genes with a p-value < 0.05 have had their p-value marked in bold. All calculations were performed using the "RT² qPCR profiler Array Data Analysis Spreadsheet" in excel.

		$2^{-\Delta\Delta Ct}$		
Gene	Fold Change	Knockdown	Scramble	p-value
ACTN1	0.83	1.5E-01	1.8E-01	0.115664
ACTN2	1.06	9.9E-06	9.4E-06	0.906610
ACTN3	2.28	1.0E-04	4.5E-05	0.964520
ACTN4	0.83	3.2E-01	3.8E-01	0.020277
AJAP1	0.87	3.7E-04	4.3E-04	0.576403
ANAPC1	1.15	1.1E-02	9.2E-03	0.110616
ARF6	0.96	2.5E-01	2.7E-01	0.399816
ARVCF	0.80	1.4E-03	1.7E-03	0.082617
BAIAP2	0.59	3.1E-03	5.2E-03	0.159858
CBLL1	1.06	4.2E-02	4.0E-02	0.304813

CDC27	0.99	2.0E-02	2.0E-02	0.867347
CDC42	0.98	1.3E-01	1.4E-01	0.809091
CDH1	1.08	2.8E-01	2.6E-01	0.449571
CDH2	0.68	2.6E-06	3.8E-06	0.199507
CDH3	0.92	5.4E-02	5.8E-02	0.402450
CDH4	1.07	6.6E-05	6.2E-05	0.633885
CDH5	0.75	4.8E-06	6.4E-06	0.669063
CDSN	1.40	1.6E-03	1.2E-03	0.046956
CSNK2A1	0.90	6.5E-02	7.2E-02	0.271377
CSNK2A2	0.77	5.2E-03	6.7E-03	0.043101
CSNK2B	1.15	8.7E-02	7.6E-02	0.119951
CTNNA1	0.94	1.8E-01	1.9E-01	0.496065
CTNNA2	0.68	2.6E-06	3.8E-06	0.199507
CTNNA3	0.46	2.8E-06	6.0E-06	0.074072
CTNNB1	0.94	2.4E-02	2.5E-02	0.608607
CTNND1	0.72	4.5E-02	6.3E-02	0.040615
DLG5	0.90	1.3E-02	1.4E-02	0.245192
DLL1	0.84	4.0E-03	4.7E-03	0.190092
DNM1	0.94	1.1E-02	1.2E-02	0.699427
DNM2	0.90	1.6E-02	1.8E-02	0.536169
DOCK4	0.84	6.0E-05	7.1E-05	0.330424
DSC1	0.81	7.6E-05	9.3E-05	0.466802
DSC2	0.96	9.2E-03	9.6E-03	0.625386
DSC3	0.86	3.3E-06	3.8E-06	0.810542
DSG1	0.32	9.7E-06	3.0E-05	0.235319
DSG2	1.07	4.6E-02	4.4E-02	0.403351
DSG3	0.48	3.0E-06	6.2E-06	0.373559
DSG4	1.82	6.9E-06	3.8E-06	0.281453
DSP	1.16	1.2E-01	1.1E-01	0.029349
EXOC2	0.88	1.5E-02	1.7E-02	0.077498
FARP2	0.88	1.2E-03	1.4E-03	0.187330
FLNA	0.94	3.7E-01	4.0E-01	0.566237
FLNB	0.83	1.7E-01	2.1E-01	0.320782
HGS	1.02	6.9E-03	6.7E-03	0.710078
IQGAP1	1.01	9.4E-02	9.4E-02	0.931711
JUP	0.83	6.6E-02	8.0E-02	0.125040
LMO7	0.87	4.2E-03	4.8E-03	0.230840
MAPRE1	1.00	9.2E-02	9.2E-02	0.952960
MAPRE2	0.89	7.8E-04	8.8E-04	0.282487
MLLT4	0.88	1.2E-02	1.4E-02	0.302713
NME1	1.31	1.4E-01	1.1E-01	0.005504

NOTCH1	0.94	5.0E-03	5.3E-03	0.583941
NOTCH2	1.07	4.2E-02	3.9E-02	0.437137
NOTCH3	0.74	2.5E-02	3.4E-02	0.136389
NOTCH4	0.85	2.4E-04	2.8E-04	0.313678
P2RX6	0.62	2.5E-05	4.1E-05	0.404917
PARD3	0.98	1.1E-02	1.1E-02	0.711133
PERP	1.23	1.4E-03	1.1E-03	0.141632
PIK3CG	0.68	2.6E-06	3.8E-06	0.199507
PKP1	0.91	1.4E-02	1.6E-02	0.301747
PKP2	1.19	1.7E-02	1.4E-02	0.043531
PKP3	0.96	4.6E-02	4.8E-02	0.764469
PKP4	0.87	4.6E-02	5.4E-02	0.061466
PNN	1.18	5.9E-02	5.0E-02	0.237154
PPAP2B	0.84	7.1E-03	8.4E-03	0.292160
PPL	0.99	5.9E-02	6.0E-02	0.962484
PVRL1	0.88	2.6E-02	2.9E-02	0.120517
PVRL2	0.89	1.3E-01	1.4E-01	0.350571
PVRL3	1.23	1.7E-03	1.4E-03	0.220280
PVRL4	0.74	1.2E-02	1.7E-02	0.153740
RAC1	1.01	2.3E-01	2.3E-01	0.806500
RALA	0.88	6.4E-02	7.3E-02	0.294289
RHOA	0.95	1.0E-01	1.0E-01	0.422377
SORBS1	1.13	7.2E-04	6.4E-04	0.263173
SSX2IP	0.92	1.1E-02	1.2E-02	0.438691
TJP1	1.13	6.8E-02	6.0E-02	0.019167
TLN1	0.90	1.6E-02	1.7E-02	0.504728
TLN2	1.04	2.8E-03	2.7E-03	0.730490
VCL	0.82	4.8E-02	5.8E-02	0.055765
VEZT	1.07	2.2E-02	2.1E-02	0.214859
WAS	0.94	3.4E-03	3.6E-03	0.658110
WASF1	1.16	8.7E-03	7.5E-03	0.003203
WASL	0.88	7.7E-03	8.8E-03	0.341148
ZYX	0.73	8.1E-02	1.1E-01	0.061866
ACTB	0.80	5.6E+00	6.9E+00	0.177284
B2M	0.90	4.9E-01	5.5E-01	0.056493
GAPDH	1.15	1.7E+00	1.5E+00	0.063450
HPRT1	1.18	6.3E-02	5.3E-02	0.057648
RPLP0	1.02	3.4E+00	3.3E+00	0.835443

Table S2. All genes screened via "human extracellular matrix and adhesion molecules" RT² qPCR array . $2^{-\Delta\Delta Ct}$ scores were calculated relative to *GAPDH* as a normalising housekeeping gene. Genes with a fold change > 2 or < 0.5 have had their fold change marked in bold. Genes with a p-value < 0.05 have had their p-value marked in bold. All calculations were performed using the "RT² qPCR profiler Array Data Analysis Spreadsheet" in excel.

		$2^{-\Delta\Delta Ct}$		
Gene	Fold Change	Knockdown	Scramble	p-value
ADAMTS1	1.77	4.5E-05	2.5E-05	0.610378
ADAMTS13	0.59	1.7E-03	2.8E-03	0.096113
ADAMTS8	0.53	1.2E-05	2.3E-05	0.492762
CD44	0.58	9.5E-02	1.6E-01	0.013010
CDH1	1.09	5.3E-01	4.9E-01	0.622432
CLEC3B	0.57	1.1E-05	2.0E-05	0.395492
CNTN1	0.75	5.1E-06	6.8E-06	0.567471
COL11A1	2.14	1.5E-05	6.8E-06	0.207364
COL12A1	0.99	1.7E-02	1.7E-02	0.843418
COL14A1	0.29	9.8E-05	3.4E-04	0.394398
COL15A1	1.21	8.2E-06	6.8E-06	0.530113
COL16A1	0.61	5.1E-04	8.5E-04	0.187912
COL1A1	0.97	4.1E-03	4.2E-03	0.938625
COL4A2	1.98	4.6E-04	2.3E-04	0.182559
COL5A1	1.30	4.0E-02	3.1E-02	0.640309
COL6A1	0.59	5.2E-03	8.9E-03	0.005121
COL6A2	0.68	2.1E-03	3.1E-03	0.335268
COL7A1	0.73	4.9E-04	6.7E-04	0.097175
COL8A1	0.58	5.1E-06	8.8E-06	0.301919
CTGF	1.64	2.1E-03	1.3E-03	0.517101
CTNNA1	0.92	2.7E-01	3.0E-01	0.759017
CTNNB1	0.92	3.6E-02	3.9E-02	0.561418
CTNND1	0.80	9.0E-02	1.1E-01	0.106207
CTNND2	1.07	8.0E-03	7.5E-03	0.953266
ECM1	1.07	2.1E-04	2.0E-04	0.999658
FN1	0.68	3.2E-02	4.6E-02	0.182809
HAS1	0.73	6.5E-05	8.9E-05	0.543897
ICAM1	0.54	3.8E-03	7.1E-03	0.009810
ITGA1	1.05	1.9E-04	1.8E-04	0.598848
ITGA2	1.12	2.2E-02	1.9E-02	0.562210
ITGA3	0.71	4.4E-02	6.2E-02	0.023212
ITGA4	0.16	1.1E-05	6.9E-05	0.033471
ITGA5	0.75	7.0E-03	9.3E-03	0.430683
ITGA6	1.38	2.4E-03	1.7E-03	0.126874

ITGA7	0.69	5.9E-04	8.6E-04	0.242675
ITGA8	0.53	5.1E-06	9.7E-06	0.234502
ITGAL	1.15	1.5E-04	1.3E-04	0.641042
ITGAM	0.86	5.0E-04	5.8E-04	0.582762
ITGAV	0.67	7.3E-02	1.1E-01	0.138623
ITGB1	0.82	2.0E-01	2.4E-01	0.033797
ITGB2	0.85	1.6E-03	1.8E-03	0.696834
ITGB3	1.27	4.1E-05	3.2E-05	0.781702
ITGB4	1.12	5.9E-02	5.2E-02	0.535322
ITGB5	0.90	5.2E-02	5.7E-02	0.517014
ANOS1	1.58	3.6E-04	2.3E-04	0.356454
LAMA1	0.72	5.1E-06	7.2E-06	0.483307
LAMA2	0.49	7.1E-05	1.5E-04	0.387150
LAMA3	0.75	4.9E-03	6.6E-03	0.394555
LAMB1	1.18	1.7E-03	1.4E-03	0.780743
LAMB3	0.41	2.6E-02	6.3E-02	0.139120
LAMC1	0.77	7.5E-02	9.7E-02	0.030582
MMP1	0.60	7.9E-06	1.3E-05	0.732275
MMP10	0.82	7.3E-06	8.9E-06	0.396895
MMP11	1.02	1.1E-03	1.1E-03	0.815091
MMP12	0.34	6.6E-04	2.0E-03	0.599603
MMP13	0.75	5.1E-06	6.8E-06	0.567471
MMP14	0.41	1.4E-04	3.6E-04	0.054865
MMP15	1.06	2.1E-04	2.0E-04	0.884209
MMP16	0.84	3.2E-03	3.8E-03	0.597330
MMP2	1.41	1.5E-05	1.0E-05	0.578603
MMP3	0.86	5.9E-06	6.8E-06	0.779029
MMP7	0.58	8.2E-06	1.4E-05	0.403380
MMP8	0.57	9.9E-06	1.8E-05	0.201549
MMP9	1.09	2.1E-03	1.9E-03	0.440664
NCAM1	0.77	3.3E-05	4.2E-05	0.666247
PECAM1	1.31	6.8E-05	5.2E-05	0.883502
SELE	0.85	1.1E-05	1.3E-05	0.804218
SELL	0.60	3.9E-03	6.5E-03	0.019988
SELP	1.91	1.3E-05	6.8E-06	0.151765
SGCE	0.82	4.2E-03	5.1E-03	0.144015
SPARC	1.14	6.8E-05	5.9E-05	0.559416
SPG7	0.83	3.7E-02	4.5E-02	0.149686
SPP1	0.48	5.1E-06	1.1E-05	0.251592
TGFBI	0.55	2.4E-02	4.3E-02	0.310831
THBS1	1.19	1.4E-01	1.2E-01	0.271260

THBS2	0.15	2.2E-05	1.4E-04	0.002481
THBS3	0.60	6.3E-03	1.1E-02	0.088154
TIMP1	0.81	1.9E-02	2.3E-02	0.107068
TIMP2	0.81	5.2E-02	6.4E-02	0.497353
TIMP3	0.44	5.9E-03	1.3E-02	0.024631
TNC	3.36	5.1E-05	1.5E-05	0.376510
VCAM1	0.75	5.1E-06	6.8E-06	0.567471
VCAN	0.75	5.1E-06	6.8E-06	0.567471
VTN	1.35	3.7E-04	2.7E-04	0.821234
ACTB	1.03	5.8E+00	5.6E+00	0.920727
B2M	0.82	4.0E-01	4.8E-01	0.526361
GAPDH	1.08	1.8E+00	1.7E+00	0.654743
HPRT1	1.16	6.5E-02	5.6E-02	0.120095
RPLP0	0.95	3.7E+00	3.9E+00	0.797525
